# Protein Adequacy in Europe: Adjusting Crude Intakes Using the Protein Adequacy and Quality Score (PAQS)

**DOI:** 10.1016/j.cdnut.2025.107539

**Published:** 2025-08-29

**Authors:** Samantha Nikita Heerschop, Sander Biesbroek, Merel Celine Daas, Anneleen Kuijsten, Mirjana Gurinović, Johanna Marianne Geleijnse, Pieter van ’t Veer

**Affiliations:** 1 Division of Human Nutrition and Health, Wageningen University & Research, Wageningen, Gelderland, The Netherlands; 2 Centre of Research Excellence in Nutrition and Metabolism, Institute for Medical Research, National Institute of Republic of Serbia, University of Belgrade, Capacity Development in Nutrition (CAPNUTRA), Belgrade, Serbia

**Keywords:** protein quality, utilizable protein, protein requirements, indispensable amino acids, PAQS, European adult population, EFSA EU Menu

## Abstract

**Background:**

Because of high overall protein intakes and the substantial proportion of animal-based protein in Europe, protein quality was not considered an issue. However, this may become important when moving toward more sustainable diets that include more plant protein.

**Objectives:**

To facilitate the assessment of protein adequacy while considering protein quality, this study aimed to provide an overview of current protein intake and adequacy in European adults, based on crude and utilizable protein.

**Methods:**

To assess the prevalence of inadequate protein intake in Europe, the Protein Adequacy and Quality Score (PAQS) was developed and applied to adults (18–64 y) of 25 national dietary surveys obtained from the Comprehensive European Food Consumption Database of the European Food Safety Authority. The PAQS assesses protein adequacy as the ratio of daily utilizable protein intake to requirement, with utilizable protein calculated on a meal basis by considering protein digestibility, indispensable amino acid requirements, and crude protein intake.

**Results:**

When accounting for energy misreporting, within-subject variation, and nonnormality of the intake distribution, the prevalence of crude protein inadequacy was <1% in both sexes. Utilizable protein inadequacy ranged from 0% (Spain) to 7% (Austria) in females, except for Germany (17%), and from 0% (Montenegro) to 6% (Germany) in males.

**Conclusions:**

These findings suggest no immediate concern for healthy adults regarding utilizable protein intake of current diets. However, diets of females in Germany may warrant closer attention.

## Introduction

Current mean crude protein intake in 20 European countries ranges between 0.8 g/kg/d and 1.25 g/kg/d [1]. These intakes largely exceed the estimated average requirement (EAR) for protein of 0.66 g/kg body weight (BW) per day (g/kg/d). Because of the high overall protein intakes in the European population, and because of the substantial proportion of animal-based protein in the diet (55%–67%), protein quality was not considered an issue so far [1]. However, to reach climate neutrality by 2050 as agreed upon in the Green Deal [2], a shift toward more sustainable diets is needed. For European and other Western countries, these diets would typically include more plant- and less animal-based foods, as the latter generally have higher environmental footprints [3,4]. As plant-based foods are also known for their lower protein quantity and quality [5], the diet shift necessitates careful evaluation of protein adequacy.

Protein intake is considered adequate when it meets physiological requirements for maintenance, growth, and development [6]. Protein that can be utilized for body protein synthesis depends on digestion and absorption as well as the extent to which indispensable amino acids (IAAs) meet the reference profile, also referred to as protein quality. To assess protein adequacy on the population level, the EAR cut-point method can be used [7], which defines the prevalence of individuals with inadequate protein intake. The EAR for protein is based on nitrogen balance studies, which considered good-quality protein. An average protein intake of 0.66 g/kg/d (EAR) is enough for 50% of the population; an intake of 0.8 g/kg/d [Recommended Dietary Allowance (RDA)] is enough for 97.5% of the population. When protein is of imperfect quality, protein intake should be increased to account for the reduced protein quality. Besides, nitrogen balance studies tend to underestimate protein requirements [8], and other methods suggest higher requirements [9]. Therefore, in this study, we assume that corrections for protein quality must be applied to the diet rather than to the requirement, which is in line with other studies [8,10]. Furthermore, as protein adequacy is directly affected by energy misreporting, which is a common problem in dietary assessment methods [11], it is important to adjust protein intake for this.

To date, most studies evaluated protein adequacy of diets by means of crude daily protein intake [[12], [13], [14]]. A few studies also assessed protein quality by either comparing daily IAA intake with their individual requirements [15,16], or by applying protein quality measures such as the Protein Digestibility-Corrected Amino Acid Score (PDCAAS) [[17], [18], [19], [20]] or the Digestible IAA Score (DIAAS) [10]. However, PDCAAS and DIAAS assess protein quality of a single food item or meal by assigning a score between 0 and 100%, indicating the percentage of the consumed protein that is utilizable per gram of consumed protein [6]. This implies that PDCAAS and DIAAS do not consider the consumed amount of protein in the diet (quantity), nor the daily protein requirement, whereas this is crucial to assess protein adequacy. Moreover, PDCAAS and DIAAS do not specify the time window in which IAAs from individual food items can complement each other. To evaluate the adequacy of protein intake, it has been argued that IAA profiles should be evaluated during the time window of a meal rather than the whole day and additionally summed over the day [21].

To facilitate the assessment of protein adequacy while considering utilizable protein intake, we integrated the PDCAAS or DIAAS in the Protein Adequacy and Quality Score (PAQS) and applied the PAQS to the Comprehensive European Food Consumption Database of the European Food Safety Authority (EFSA). The PAQS assesses protein adequacy as the ratio of daily utilizable protein intake to requirement. Daily utilizable protein intake is calculated as the sum of utilizable protein intake per meal after considering protein digestibility, IAA and dispensable amino acid (DAA) requirements, and crude protein intake. The aim of this study was to provide an overview of current protein intake and adequacy in European adults, based on crude or utilizable protein, using individual-level food consumption data including information on meal moments and adjusted for misreporting.

## Methods

### Study population and dietary assessment

Individual-level national food consumption data from 28 European countries, collected between 2003 and 2021 via multiple 24-h dietary recalls or (web-based) food records, were obtained from the EFSA Comprehensive European Food Consumption Database [22], with country permissions. This includes new dietary surveys from the Balkan region, including Bosnia and Herzegovina [23], Montenegro [24], and Serbia [25]. Methodological details are available via original country publications and the EFSA database (Supplemental Table 1). Nineteen countries followed the EU Menu methodology [26], which requires 2 nonconsecutive 24-h dietary recalls using Computer-aided interviews (CAPI)/Computer-aided telephone interviews (CATI) methods, usage of the EFSA FoodEx2 food classification system [27], and collection of weight, height, and physical activity data [26]. Information on meal moments was available in all countries, except the United Kingdom. The number of recorded consumption days per individual ranged from 1 to 7 (Supplemental Table 2).

Food consumption data were available for 81,202 participants. Countries with only 1 measurement day were considered in the baseline data but excluded from further analyses (Poland, Bulgaria, Slovakia: *n* = 8099) because of insufficient data for estimating usual intake. Children (<18 y) and older adults (>64 y) (*n* = 31,159), lactating, and pregnant females (*n* = 1364) were excluded. Finally, participants with implausible energy intakes (*n* = 1038), defined as participants in the lowest and highest 1.25% of reported energy intakes in each country, were excluded. The remaining study population consisted of 39,542 participants.

### Food composition

Because several countries lacked country-specific food composition tables, the Dutch Food Composition Database was used to estimate energy and protein intake for all countries (NEVO-online version 2021/7.1). Food consumption data were linked to NEVO through the Foodex2 classification system [27]. A detailed description of the linking process is described elsewhere [28]. Because the Dutch Food Composition Database lacks amino acid (AA) composition and protein digestibility data, the AA database V2.0 developed by Wageningen University and Research was used, as well as fecal protein digestibility factors per food group as previously reported [19]. As the sum of AAs in a product did not always correspond to the total amount of protein in that product, we normalized the AAs so that their sum did correspond to the total amount of protein.

### Calculation of the PAQS

Daily protein intake is adequate once the sum of utilizable protein across meals equals or exceeds the requirement. Utilizable protein refers to AAs that are bioavailable from the diet. Utilizable or bioavailable AAs are those that match the reference IAA profile after digestion and absorption. In this study, we applied the conversion of crude to utilizable protein to the diet rather than to the requirement, as the EAR for protein is based on good-quality protein, and may underestimate actual needs [8,9]. Protein adequacy involves 3 components:

(1) meeting the requirements for the IAAs according to the reference profile,
(2) meeting the requirements for the DAAs, that is, the appropriate ratio of IAAs and DAAs, and
(3) meeting the daily protein (nitrogen) requirement.

The first 2 components relate to protein quality and are derived from protein digestibility and AA reference profiles [6]. The third component refers to protein quantity and includes the crude protein intake. Protein quality should be evaluated at the meal level, because AAs from different products may complement each other within a meal [21,29], and it is unclear yet whether this occurs across meals [30]. In this study, protein adequacy was calculated according to the PAQS, which expresses all components *1*), *2*), and *3*) [formula (1)] (Supplemental Methods 1)

(1) $$\text{PAQS}=\sum_{m}\left(\mathrm{MIN}\left(\frac{\sum_{j}{\text{AIAA}}_{bjm}}{R_{b}},\frac{\sum_{cj}{\text{ADAA}}_{cjm}}{\text{RDAA}},\frac{\text{ATA}A_{m}}{\mathrm{PR}}\right)\right)$$

where ${\text{AIAA}}_{bjm}$ is the absorbed IAA *b* from food item *j* from meal moment *m* in mg/kg. $R_{b}$ is the required amount of utilizable IAAs *b* in mg/kg/d. ${\text{ADAA}}_{cjm}$ is the absorbed DAA *c* from food item *j* from meal moment *m* in mg/kg. RDAA is the required amount of utilizable DAAs in mg/kg/day. $A\text{TA}A_{m}$ is the total absorbed AAs in meal moment *m* in mg/kg, that is, the sum of the absorbed IAAs and DAAs. PR is the required total amount of utilizable IAAs and DAAs in g/kg/day [6].

In words, the first ratio in formula (1) calculates the absorbed IAAs (${\text{AIAA}}_{bjm}$) in a meal relative to body requirements ($R_{b}$), that is, the AA reference profile. The absorbed IAAs are obtained as the product of AA intake and protein digestibility. This first ratio is comparable to the PDCAAS or DIAAS, depending on the type of digestibility factor used. The second ratio calculates the absorbed DAAs ($\text{ADA}A_{cjm}$) relative to body requirements (RDAA). RDAA is defined as the daily protein requirement minus the sum of the IAA requirements ($\mathrm{PR}-{\sum_{b}R}_{b}$). The last ratio calculates the total of absorbed IAAs and DAAs relative to the total daily protein requirement (PR). Then, the minimum of the 3 ratios is taken and summed over the meals of the day. This way, the PAQS is the meal-based ratio of utilizable to required protein and should exceed 1 for protein adequacy.

For the calculations, we have used the EARs for AAs and protein for adults (aged >18) as defined by the FAO of the United Nations [6]. In this study, it is assumed that all IAAs that exceed their requirement (first ratio in formula (1) >1) as set by the limiting IAA (lowest ratio of the IAAs over their requirement) can be converted to DAAs. Therefore, DAAs will never be limiting (second ratio in formula (1)). Ideally, digestibility is based on AA-specific true ileal digestibility (TID). Because such detailed data are still lacking for the full range of products, we used estimates of fecal protein digestibility instead. This implies that the TID is assumed to be the same for all IAAs *b* from food item *j*, and DAAs *c* from food item *j*. A detailed description of the PAQS can be found in Supplemental Methods 1.

In addition, 2 sensitivity analyses were performed. First, the sensitivity of the PAQS to consider separate meals was investigated by leaving meal moments out of the calculation, meaning that the IAA profile was considered over the whole day and the index *m* cancels from formula (1). This analysis was called the “Daily IAA profile” scenario. In the second analysis, the sensitivity of the PAQS to the IAA reference profile was evaluated by omitting this calculation step in the “digestibility only” scenario, meaning that only the third ratio of formula (1) was applied.

### Assessment of energy misreporting and correcting protein intake

Basal metabolic rate was calculated using the Schofield equation [31,32]. Under- and over-reporters were identified using Goldberg cut-offs [33,34]. These cut-offs were adjusted for the number of measurement days and assumed a low-to-moderate physical activity level (PAL) (1.4). According to this method, 4.7%–47.5% of participants were identified as under-reporters of energy intake. Instead of excluding these subjects, we assumed that misreporting was not differential for protein intake, and we adjusted intake for the degree of misreporting by means of a linear mixed model. In this model, protein intake (g/kg/d) was the dependent variable, and the ratio of reported energy intake over required energy intake (based on the Schofield equation with a PAL of 1.4) was the independent variable. The model included random intercepts and slopes to account for within-person variation across multiple measurement days, and was adjusted for sex, country, and BMI (in kg/m^2^). An exponential covariance structure (continuous-time spatial autocorrelation) accounted for the consecutive and nonconsecutive measurement days (Supplemental Table 1). Protein intake was predicted at an energy intake equal to energy requirement (ratio = 1), with residuals added to predicted values. Four models were developed for: *1*) crude protein intake, *2*) utilizable protein intake, *3*) daily IAA profile scenario, and *4*) digestibility only scenario. Models 1 and 2 answered the research question whereas models 3 and 4 were sensitivity analyses. Supplemental Figure 1 visualizes the distribution of utilizable protein intakes, the ratio of reported energy intake over required energy intake, and the predicted values. Normality of residuals, normality of the random effects, and homoscedasticity were graphically evaluated. There was no multicollinearity between the independent variables. The model was fitted using the nlme package version 3.1.164 from R version 4.4.1 (2024-06-14 Universal C Runtime on Windows).

### Defining the prevalence of inadequate protein intake

We used the Statistical Program to Assess usual Dietary Exposure (SPADE, v4.1.35) to estimate usual intakes of crude and utilizable protein and to estimate the prevalence of inadequacy. SPADE is an R-based software package that models usual intake distributions based on repeated 24-h dietary recall data by *1*) transforming data to a normal distribution, *2*) removing within-person variability, *3*) estimating usual nutrient intake distributions as a function of age, and *4*) back-transforming the data onto its original scale [35]. Consequently, SPADE applies the EAR cut-point method to define the prevalence of inadequacy. The 1-part model, suitable for nutrients consumed daily, was used. The subgroup-and-bootstrapping function (200 rounds) from the SPADE package was applied for each country and sex. As data for France only included age groups, that is, adults aged 18–64, SPADE modeled intake as a constant. The SPADE outputs used in this study were the population percentage below the EAR and 95% confidence intervals by sex.

## Results

### Baseline characteristics

The median age in the 28 national dietary surveys ranged from 33 y in Bulgaria to 47 y in Romania, and the proportion of females ranged from 49% in Hungary to 66% in Estonia (Table 1). BMI ranged from a median of 23.8 in Austria to 27.2 in Greece and median daily energy intake for most countries was around 1900–2000 kcal, with intakes lower than 1700 kcal for Bosnia and Herzegovina, Cyprus, Estonia, Portugal, Romania, Slovakia, Slovenia, and Spain, and intakes higher than 2500 kcal for Poland. The countries with low energy intakes also had relatively high proportions of under-reporters (25.0%–47.5%). Poland had the highest crude protein intake (95 g/d). Estonia and Slovakia had the lowest crude protein intake (65 g/d). Crude plant protein intake was roughly similar for all European countries (Table 1, Supplemental Figure 2). The variation in total crude protein intake between countries was primarily attributable to the amount of animal protein. The median crude protein intake in g/kg/d ranged from 1.3 times the EAR (0.66 g/kg/d) in Estonia, Germany, and Greece to 2.1 times the EAR in Poland. When utilizable protein was calculated according to the PAQS, the lowered medians ranged between 1.1 (Germany) and 1.8 times (Poland) the EAR. Countries that had a higher crude protein intake also had a higher utilizable protein intake (Table 1, Supplemental Information 5).

**TABLE 1:** Baseline characteristics of the study population, by country, derived from multiple 24-h dietary recalls or food records from 39,542 subjects available in the harmonized Comprehensive European Food Consumption Database of the European Food Safety Authority^1^.

Country (period of data collection)	Age	Sex	Weight	BMI	Energy	Crude protein			Crude plant protein	Crude animal protein	Utilizable protein	Under-reporting^2^	Over-reporting^2^
	Y, median (IQR)	Females *n* (%)	kg, median (IQR)	kg/m^2^, median (IQR)	kcal, median (IQR)	g, median (IQR)	En%, median (IQR)	g/kg BW, median (IQR)	g/kg BW, median (IQR)	g/kg BW, median (IQR)	g/kg BW, median (IQR)	%	%
Austria (2014–2018)	37 (28–48)	1396 (64)	69.5 (60.2–81.2)	23.8 (21.4–26.7)	1902 (1509–2368)	69 (54–90)	14.6 (12.3–17.3)	1.00 (0.78–1.27)	0.36 (0.27–0.47)	0.61 (0.43–0.85)	0.82 (0.62–1.07)	14.4	1.0
Belgium (2014–2015)	40 (28–52)	604 (50)	74.0 (64.1–85.6)	25.1 (22.4–28.3)	1842 (1452–2318)	74 (59–95)	16.4 (14.0–19.1)	1.02 (0.79–1.29)	0.32 (0.24–0.42)	0.66 (0.50–0.90)	0.85 (0.65–1.09)	22.6	0.6
Bosnia Herzegovina (2017–2020)	40 (27–51)	435 (52)	77.0 (66.0–90.0)	25.2 (22.8–27.8)	1617 (1359–1959)	72 (56–89)	17.4 (15.0–20.1)	0.92 (0.74–1.14)	0.31 (0.26–0.39)	0.60 (0.43–0.80)	0.79 (0.62–0.99)	33.1	0.0
Bulgaria^3^ (2004)	33 (24–51)	350 (52)	70.5 (60.0–80.0)	24.5 (21.6–27.9)	1774 (1280–2393)	66 (45–94)	14.8 (12.2–17.8)	0.94 (0.65–1.34)	0.39 (0.26–0.54)	0.54 (0.30–0.82)	0.82 (0.54–1.18)	24.7	0.9
Croatia (2011–2012)	41 (29–52)	986 (51)	75.0 (65.0–86.0)	24.9 (22.5–27.8)	1811 (1386–2335)	80 (61–102)	17.6 (15.4–20.1)	1.07 (0.83–1.37)	0.33 (0.24–0.43)	0.72 (0.53–0.95)	0.92 (0.70–1.18)	29.6	1.2
Cyprus (2014–2017)	36 (26–53)	130 (51)	73.2 (60.9–87.3)	25.2 (22.4–29.4)	1584 (1298–2049)	79 (59–105)	19.6 (16.2–22.8)	1.08 (0.82–1.39)	0.32 (0.22–0.40)	0.76 (0.52–1.04)	0.95 (0.69–1.20)	35.0	0.0
Czech Republic (2003–2004)	45 (32–55)	853 (53)	75.0 (65.0–85.0)	25.2 (22.8–27.9)	2281 (1673–2958)	82 (61–106)	14.3 (12.6–16.5)	1.11 (0.84–1.40)	0.41 (0.30–0.53)	0.69 (0.49–0.90)	0.93 (0.70–1.20)	12.6	6.2
Denmark (2005–2008)	44 (34–54)	886 (54)	74.0 (65.0–85.0)	24.4 (22.3–27.0)	2077 (1730–2540)	79 (65–98)	15.3 (13.8–16.9)	1.07 (0.88–1.31)	0.37 (0.30–0.45)	0.70 (0.56–0.88)	0.90 (0.73–1.11)	16.4	2.6
Estonia (2013–2015)	42 (30–53)	1339 (66)	75.0 (63.8–86.5)	25.5 (22.4–29.1)	1586 (1229–2015)	65 (50–85)	16.4 (13.9–19.5)	0.88 (0.66–1.15)	0.25 (0.19–0.34)	0.61 (0.43–0.83)	0.74 (0.55–0.99)	34.5	0.1
Finland (2017)	46 (34–56)	635 (51)	78.0 (67.0–90.0)	26.4 (23.4–30.0)	1950 (1585–2340)	82 (67–103)	17.1 (14.8–20.0)	1.07 (0.84–1.34)	0.30 (0.23–0.40)	0.75 (0.56–1.00)	0.92 (0.71–1.16)	21.4	0.6
France^4^(2014–2015)	— (—)	1105 (57)	70.2 (61.0–81.4)	24.6 (21.9–27.8)	1984 (1586–2488)	83 (67–102)	16.6 (14.2–19.6)	1.18 (0.92–1.48)	0.36 (0.27–0.48)	0.80 (0.60–1.03)	1.00 (0.78–1.26)	12.9	3.0
Germany (2007)	44 (35–53)	5610 (56)	74.0 (69.0–85.0)	25.7 (23.9–26.9)	1967 (1550–2472)	66 (51–84)	13.4 (11.5–15.7)	0.88 (0.68–1.11)	0.34 (0.26–0.44)	0.52 (0.37–0.70)	0.71 (0.54–0.92)	19.6	1.7
Greece (2014–2016)	42 (30–53)	126 (50)	78.2 (65.8–90.6)	27.2 (23.9–30.8)	1723 (1369–2150)	67 (52–87)	15.9 (13.4–18.2)	0.88 (0.68–1.14)	0.27 (0.19–0.36)	0.57 (0.42–0.81)	0.76 (0.57–0.99)	34.5	0.8
Hungary (2018–2020)	46 (35–56)	240 (49)	79.0 (67.0–92.0)	26.4 (23.5–30.4)	2083 (1686–2580)	83 (67–102)	15.9 (13.6–18.6)	1.07 (0.83–1.33)	0.37 (0.29–0.47)	0.69 (0.51–0.88)	0.92 (0.69–1.14)	13.5	0.8
Ireland (2008–2010)	40 (28–50)	625 (50)	76.3 (66.4–86.7)	26.3 (23.6–29.2)	2015 (1607–2509)	85 (68–105)	16.7 (14.4–19.3)	1.11 (0.90–1.35)	0.35 (0.28–0.45)	0.74 (0.57–0.93)	0.94 (0.76–1.17)	19.2	0.7
Italy (2018–2020)	41 (32–51)	369 (52)	71.0 (60.0–81.8)	24.3 (22.0–27.3)	1834 (1504–2214)	79 (63–98)	17.2 (14.7–20.3)	1.11 (0.88–1.40)	0.34 (0.27–0.44)	0.75 (0.56–1.01)	0.94 (0.74–1.21)	13.5	0.0
Latvia (2012–2015)	40 (27–51)	638 (54)	75.0 (65.0–88.0)	25.3 (22.3–28.7)	1929 (1481–2449)	78 (58–101)	16.0 (13.6–19.0)	1.03 (0.77–1.33)	0.28 (0.21–0.37)	0.74 (0.52–0.99)	0.88 (0.64–1.15)	21.5	2.0
Montenegro (2017–2021)	38 (27–51)	345 (50)	80.0 (66.0–90.0)	24.7 (22.2–27.7)	1731 (1398–2085)	77 (59–95)	17.6 (15.2–20.3)	0.97 (0.79–1.21)	0.34 (0.27–0.42)	0.61 (0.47–0.82)	0.82 (0.65–1.05)	27.7	0.0
Netherlands (2012–2016)	38 (26–50)	734 (51)	78.0 (68.0–90.0)	25.1 (22.2–28.7)	2068 (1685–2554)	80 (64–99)	15.3 (13.3–18.0)	1.01 (0.81–1.28)	0.37 (0.29–0.49)	0.61 (0.46–0.82)	0.83 (0.66–1.06)	14.4	1.5
Poland^3^ (2000)	41 (29–50)	1304 (54)	71.9 (61.8–81.4)	24.9 (22.4–28.2)	2563 (1939–3381)	95 (68–136)	14.9 (12.2–18.1)	1.38 (0.96–1.86)	0.51 (0.38–0.68)	0.82 (0.50–1.23)	1.16 (0.78–1.59)	4.7	11.4
Portugal (2015–2016)	43 (32–53)	1710 (53)	72.0 (62.0–83.0)	26.3 (23.1–29.7)	1695 (1326–2163)	87 (64–114)	20.3 (16.9–24.5)	1.19 (0.88–1.60)	0.31 (0.22–0.41)	0.87 (0.61–1.23)	1.02 (0.74–1.39)	28.2	1.1
Romania (2019–2020)	47 (36–55)	422 (51)	78.0 (65.0–90.0)	26.3 (23.4–29.6)	1453 (1074–1933)	70 (50–95)	19.0 (15.5–22.6)	0.91 (0.64–1.26)	0.29 (0.19–0.42)	0.61 (0.40–0.89)	0.79 (0.53–1.10)	47.5	0.5
Serbia (2019–2020)	42 (29–52)	568 (51)	75.2 (64.0–87.0)	24.8 (22.2–27.8)	1807 (1426–2314)	81 (64–105)	17.8 (15.0–21.2)	1.09 (0.86–1.39)	0.37 (0.28–0.49)	0.71 (0.50–0.94)	0.92 (0.70–1.18)	23.7	0.9
Slovakia^3^ (2008)	36 (29–48)	1349 (50)	75.0 (63.0–85.0)	25.0 (22.4–28.0)	1674 (1252–2274)	65 (47–87)	15.3 (12.6–18.6)	0.88 (0.64–1.19)	0.37 (0.26–0.50)	0.47 (0.30–0.73)	0.72 (0.51–0.99)	25.0	1.8
Slovenia (2017–2018)	46 (33–56)	192 (52)	77.0 (65.0–88.0)	26.2 (23.1–29.0)	1572 (1241–2011)	70 (51–89)	17.2 (14.6–20.9)	0.92 (0.65–1.24)	0.32 (0.22–0.42)	0.59 (0.38–0.86)	0.77 (0.53–1.06)	40.1	0.3
Spain (2013–2015)	40 (30–52)	269 (50)	72.0 (62.0–84.2)	25.0 (22.7–28.0)	1415 (1174–1687)	67 (57–81)	19.1 (16.4–21.9)	0.93 (0.75–1.15)	0.25 (0.19–0.33)	0.67 (0.52–0.86)	0.80 (0.64–1.02)	47.0	0.0
Sweden (2010–2011)	44 (32–54)	715 (55)	75.0 (65.0–84.3)	24.9 (22.6–27.1)	2003 (1645–2448)	88 (72–107)	17.5 (15.4–20.2)	1.20 (0.98–1.45)	0.34 (0.26–0.43)	0.84 (0.65–1.05)	1.04 (0.83–1.25)	19.5	1.7
United Kingdom (2008–2011)	41 (30–52)	690 (56)	75.2 (65.2–86.6)	26.6 (23.3–29.9)	1793 (1448–2205)	68 (55–83)	15.0 (13.2–17.4)	0.90 (0.72–1.12)	0.34 (0.26–0.43)	0.55 (0.41–0.72)	0.77 (0.61–0.96)	28.2	0.5

Abbreviations: BW, body weight; SPADE, Statistical Program to Assess usual Dietary Exposure.

1 Nutrient intakes are expressed per day.

2 Under and overreporting were defined according to the Goldberg method [33].

3 Food consumption data from Bulgaria, Poland, and Slovakia included only 1 measurement d/individual. In addition, the Polish data were obtained from multiple people within a household, resulting in interdependencies among the data. Therefore, habitual protein intake distributions of these countries could not be defined using the statistical software SPADE and they are left out of further analyses.

4 France did not provide detailed data on age and defined all subjects in the age range 18–64 y as adults.

### Protein adequacy

**FIGURE 1:**
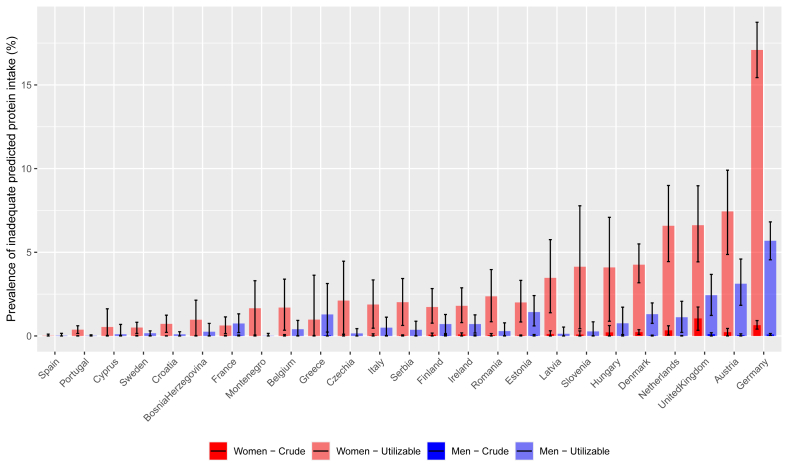
Prevalence of inadequate protein intake among adults (aged 18–64) by country, stratified by sex, using either crude or utilizable protein intake, adjusted for energy misreporting. Data were derived from multiple 24-h dietary recalls or food records from 39,542 subjects available in the harmonized Comprehensive European Food Consumption Database of the European Food Safety Authority. Daily utilizable protein intake was calculated using the Protein Adequacy and Quality Score (PAQS), by taking the sum of utilizable protein intake per meal after considering amino acid digestibility, amino acid requirements, and crude protein intake. Crude and utilizable protein intake were adjusted for energy misreporting by means of a linear mixed model, with protein intake (g/kg/d) as the dependent variable, and the ratio of reported over required energy intake as independent variable (detailed description in Methods). Protein intake was predicted from this model at energy balance, that is the ratio of reported over required energy intake was equal to 1, and assuming a physical activity level of 1.4. Prevalences were estimated by the Statistical Program to Assess usual Dietary Exposure (SPADE), which models usual intake distributions and removes within-person variation, and subsequently applies the EAR cut-point method using the EAR of 0.66 g/kg/d. Data used to generate this figure, as well as the prevalences of inadequate protein intake based on observed protein intake can be found in Supplemental Table 6.

Protein intakes underlying Figure 1 are corrected for energy misreporting by adjusting protein intake (both crude and utilizable) to the level of energy balance. The prevalence of inadequacy when considering crude predicted protein intake was a maximum of 1.0% in females (United Kingdom), and 0.1% in males (Austria, Germany, and United Kingdom). The prevalence of inadequacy based on predicted utilizable protein intake for females ranged from 0% in Spain to 7% in Austria, with an exception for Germany (17%), whereas for males this ranged from 0% in Montenegro to 6% in Germany. In 22 out of 25 countries, protein inadequacy in females exceeded that of males (Figure 1). When deriving the prevalence of inadequacy for crude protein from the observed data (unadjusted for energy misreporting), the mean prevalence of inadequacy over the 25 national dietary surveys was 6.6% (Supplemental Table 6).

### Sensitivity analyses

**FIGURE 2:**
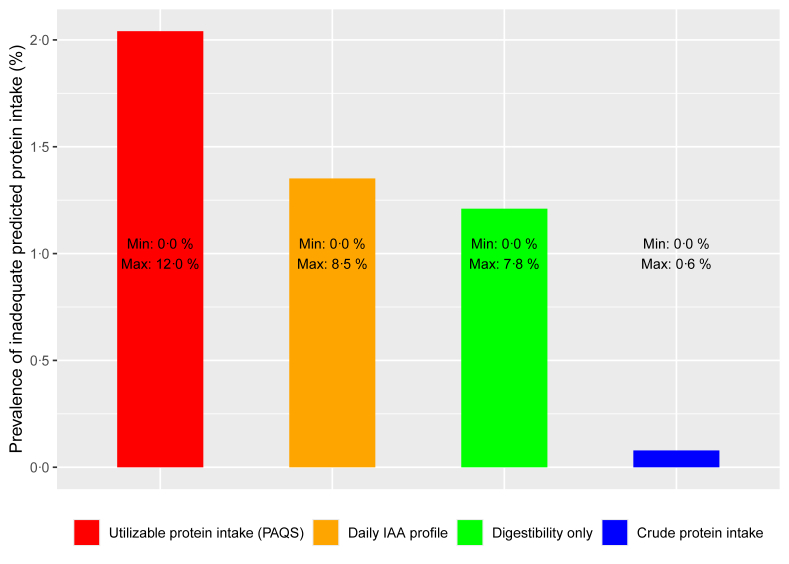
Mean and the range (min, max) of 25 country-specific prevalences of inadequate protein intake (%) among adults (aged 18–64), adjusted for energy misreporting. Data were derived from multiple 24-h dietary recalls or food records from 39,542 subjects available in the harmonized Comprehensive European Food Consumption Database of the European Food Safety Authority. Utilizable protein intake [Protein Adequacy and Quality Score (PAQS)]: protein intake was calculated using the PAQS, by taking the sum of utilizable protein intake per meal after considering protein digestibility, amino acid requirements, and crude protein intake. Daily IAA profile: considering digestibility, the daily IAA profile, that is, meal moments were not considered when defining the limiting IAA, and crude protein intake; digestibility only: crude protein intake that is adjusted for digestibility. For all scenarios, the prevalence of inadequacy is calculated as described under Figure 1. IAA, indispensable amino acid.

Because of the scientific uncertainty on the exact time window in which IAAs can complement each other, we performed a sensitivity analysis where the IAA profile was considered at the day level, meaning that meal moments were not considered when defining the limiting IAA. In this “daily IAA profile” scenario, complementary IAAs can be combined throughout the day, which increases the calculated protein quality and thereby the apparent protein adequacy. As a result, the prevalence of inadequate predicted protein intake, as mean of the 25 country-specific prevalences, was lower than when using the PAQS (Figure 2). In the “daily IAA profile” scenario, the mean proportion of measurement days with a limiting AA was 2.9% (data not shown), meaning that on most measurement days the IAA profile was complete. In case there was still a limiting AA, this was mainly lysine (data not shown).

Furthermore, the sensitivity of the PAQS to the IAA reference profile was evaluated by omitting this calculation step in the “digestibility only” scenario. As expected, the prevalence of inadequate predicted protein intake was only slightly lower in the “digestibility only” scenario than in the “daily IAA profile” scenario: in case, the IAA profile is not limiting, utilizable protein intake is defined by the amount of protein that can be digested and absorbed, leading to the “digestibility only” scenario (Figure 2).

### Limiting AAs

**FIGURE 3:**
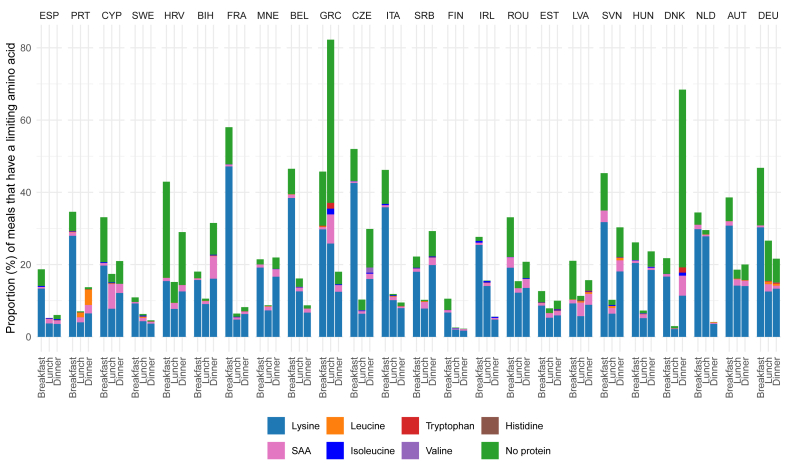
Proportion (%) of meals with a limiting amino acid, or not containing any protein, by meal moment and country. Breakfast, lunch, and dinner accounted for a mean of 82% of total protein intake. In-between meals are left out from this figure. The aromatic amino acids and threonine were never limiting and therefore not shown in this graph. The United Kingdom is not included in this graph because data on meal moments were not available. Data for Greece and Denmark should be interpreted with caution, as there is likely a meal coding issue (Supplemental Table 7). AUT: Austria; BEL: Belgium; BIH: Bosnia and Herzegovina; CYP: Cyprus; CZE: Czech Republic; DEU: Germany; DNK: Denmark; ESP: Spain; EST: Estonia; FIN: Finland; FRA: France; GBR: United Kingdom; GRC: Greece; HRV: Croatia; HUN: Hungary; IRL: Ireland; ITA: Italy; LVA: Latvia; MNE: Montenegro; NLD: Netherlands; PRT: Portugal. ROU: Romania; SAA: Sulfur amino acids. SRB: Serbia; SVN: Slovenia; SWE: Sweden. Data used to generate this figure can be found in Supplemental Table 9. Some food items were not categorized as part of a meal and were instead labeled as “Unclassified.” These food items are left out of this figure, and their contribution to crude protein intake is shown in Supplemental Table 7.

The protein quality of a meal is <100% when there is a limiting AA in a meal, which reduces protein adequacy. Lysine was the main limiting AA in all countries, followed by the sulfur AAs (SAA) (Figure 3). In most countries, breakfasts had the highest total proportion of limiting AAs, with lysine being the primary limiting AA. However, in Serbia, lysine was most frequently limiting during dinner. In Greece, lunch had the highest proportion of limiting AAs, and in Bosnia and Herzegovina, Latvia, Denmark, and Serbia dinner had the highest proportion. During lunch in Greece and dinner in Denmark, the percentage of meals without protein was high. This is likely because of inconsistent meal coding, as observed from the contribution of crude protein intake per meal to the daily protein intake (Supplemental Table 7). Therefore, results for Greece and Denmark should be interpreted with caution. Daily IAA intake is shown in Supplemental Table 8.

## Discussion

This study provides estimates of the intake and inadequacy of crude and utilizable protein across 25 European countries. We showed that the median crude protein intake in Europe largely exceeds the EAR (1.3–2.1 times higher). When applying the EAR cut-point method after adjusting for energy misreporting, within-subject variation, and nonnormality of the intake distribution, the maximum prevalence of crude protein inadequacy was 1.0% for females in the United Kingdom and 0.1% for males in Austria, Germany, and the United Kingdom. For utilizable protein, that is, after applying the PAQS, inadequacy ranged from 0% (Spain) to 7% (Austria) in females, with the exception of Germany (17%), and 0% (Montenegro) to 6% (Germany) in males. These findings suggest that with the current diet composition and quantity of protein intake there is no cause for concern in the healthy adult population, with a possible exception for females in Germany.

In a global study, the ratio of protein intake over PR was assessed by per capita protein intake corrected for the DIAAS [10], whereas in the United States individual consumption data (NHANES dataset) were corrected for theoretical DIAAS values, and prevalence of protein inadequacy was estimated [36]. Compared with these studies, the present study estimates the prevalence of protein inadequacy in 25 European countries based on the PAQS: a standardized score to assess utilizable protein intake relative to requirements. Furthermore, the present study uses harmonized individual-level food consumption data, adjusts for energy misreporting and applies the EAR cut-point method to estimate prevalences of inadequacy. In the NHANES study, 10.6% of the adults (19–50 y) had inadequate crude protein intake based on the EAR cut-point method (36). This is comparable to the mean European prevalence of 6.6% found in this study, based on the observed food consumption data (Supplemental Table 6). However, we showed that this level of inadequacy is largely attributable to underreporting of overall food intake as the mean prevalence of inadequate crude protein intake dropped to nil upon adjustment for underreporting (Figure 2). The prevalence of underreporting for part of the NHANES data was estimated at 25% [37], which aligns with the prevalence in the countries of this study.

In most countries, higher protein inadequacy was observed among females than males. This difference may reflect variations in dietary patterns (what they eat) and energy (how much they eat) between females and males. It could also be attributed to the current scaling of protein requirements, which is based on BW (kg). It has been suggested that protein requirements might be more accurately estimated based on body composition rather than BW [38], because of differences in fat-free mass (FFM) between males and females, young and older, active and inactive, and underweight and overweight persons [39]. Redefining protein requirements may lower inadequacy rates in females because of lower FFM, and raise them in males because of higher FFM. However, to date, it is unclear what the actual protein requirements per kg FFM are for different target groups [39]. The higher inadequacy among females than males was more extreme among German females (17.1%) than other European females (0%–7.4%) (Figure 1). This may be because of dietary patterns, as German females consume less bread, cereals, potatoes, and pastries, and about half the amount of meat, fish, and eggs compared with males [40]. Because protein quality is largely determined by the limiting AA, identifying it can help to improve the protein quality of meals. The primary limiting AA in this study, lysine, is limiting in cereals [5]. This highlights the presence of cereals as a staple food group in European diets [41,42]. The second limiting AA, though much less frequent than lysine, is the group of SAAs, which are limiting in legumes. This highlights the much lower consumption of legumes in European diets compared with cereals [41].

Although the PAQS assumes that IAAs can only complement each other within meals, there is no scientific consensus yet about this time window. Some experts suggest that this complementation is ideally achieved within a single meal, particularly in cases of lower protein diets or when specific IAAs are deficient on consecutive days [29], whereas others advocate that complementation can be spread over a day [30]. The best estimate of the prevalence of inadequacy likely falls in between 2.0% (when using the PAQS) and 1.4% (based on the “Daily IAA profile” scenario). Note that for the United Kingdom no data on meal moments were available. Therefore, the “utilizable” and the “Daily IAA profile” scenario gave identical results (data not shown), and the prevalence of inadequacy of utilizable protein intake will be slightly underestimated. As the IAA profile in the “Daily IAA profile” scenario is nearly always complete, the prevalence of inadequacy for the “Digestibility only” scenario (1.2%) is only slightly lower than that of the “Daily IAA profile” scenario (1.4%). Therefore, if the consensus establishes that “the time window for amino acids to be complementary is one day,” utilizable protein mainly depends on the quantity of digestible protein. Consequently, research efforts can prioritize studying protein digestibility rather than defining IAA profiles of food items.

The PAQS evaluates protein adequacy based on utilizable protein intake in populations, whereas other recent measures of protein quality and quantity focus on individual diet planning essential amino acid score 9 (EAA-9) methods [43], and planning and evaluation at the meal level [Meal Protein Quality Score (MPQS)] [44] (Supplemental Methods 1). The PAQS calculates utilizable protein on a per-meal basis, and consequently sums it over the day. As the PAQS aims to evaluate protein adequacy on the population level, the EAR for protein is used, which differs from the EAA-9 that is designed for individual-level meal planning and uses the RDA as target. Besides, the PAQS compares daily utilizable protein intake with the daily protein requirements (EAR), whereas the MPQS uses meal protein (0.3 g/kg) and AA requirements for older adults to allow for guidance in meal planning. An additional characteristic of the PAQS compared with the EAA-9 and the MPQS is that the PAQS can correct for impaired conversion of IAAs to DAAs, potentially making DAAs the limiting factor rather than IAAs [45,46]. For example, it has been reported that lysine and threonine do not undergo transamination because the primary group on the side chain of lysine and the hydroxyl group on the side chain of threonine prevent enzymatic transamination [47]. When the aim is to assess protein adequacy, the established protein quality scores PDCAAS and DIAAS are not suitable in their current form, because they lack quantity aspects, guidance on how to cope with meal moments, and they do not consider the potentially limiting DAAs. Although in our analyses, DAAs where in practice never limiting adding the term to PAQS is correct. In potential scenarios, even if the IAA profile is correct and the intake recommendations for the individual IAAs are met, a deficiency in total protein intake will still result in some IAAs being metabolized to DAAs. Because this is at the expense of the IAAs, the requirement would then not be met. In contrast, the PAQS provides a clear and standardized formula that can directly be applied to dietary intake data, which facilitates application in the field and allows for consistent interpretation: that is, utilizable protein is calculated first per meal, then summed over the day, and no calculation steps additional to this formula are needed to assess protein adequacy. Note that to obtain the percentage of the population with inadequate protein intake according to the PAQS, methods like the NCI method or SPADE can be applied to correct for day-to-day variability within subjects by using PAQS = 1 as the requirement [35,48,49]. These methods estimate usual intake distributions and subsequently use the EAR cut-point method to define the adequacy of a population (Supplemental Methods 1). Because the PAQS is a linear score, it can be applied as a constraint or an objective function in diet optimization studies that use linear programming [50], to monitor protein adequacy in detransition toward sustainable diets. As current European protein intakes largely exceed the requirement and the contribution of animal protein is substantial (55%–67%) [1], initial dietary adjustments toward sustainable diets are no reason for concern of widespread inadequate protein intakes, as was also shown for the Dutch population [19].

As other studies in this domain, this study was confronted with several data quality issues, such as the sparse availability of country-specific AA compositions and AA digestibility data, the use of different dietary assessment methods (24-h dietary recalls or food records) and energy misreporting linked to these methods, uncertainties on the validity of IAA and protein requirements, and suitable scaling of protein requirements. The lack of detailed digestibility data supports the relevance and importance of a unified digestibility database including high-quality digestibility data of all existing foods [51]. The adjustment for energy misreporting that we performed covers the potential differential extent of misreporting between 24-h dietary recalls and food records. In case the true PAL is lower than the assumed 1.4, the current ratio of reported over required energy intake is underestimated, protein intake is overestimated, and the prevalence of inadequacy is underestimated. In case of higher PALs, the current prevalence of inadequacy is overestimated. Current protein requirements may be underestimated, as they are based on nitrogen balance studies, which tend to overestimate nitrogen intake because of unmeasured wastage and spillage of ingested protein, and are suggested to underestimate nitrogen losses because of inaccuracies in collecting excreta and quantifying miscellaneous losses [8]. In addition, there are further uncertainties on the assumed linearity between nitrogen intake and nitrogen balance, which affects the interpretation of positive nitrogen balance and thereby the requirement. Similarly, IAA requirements tend to be underestimated because of theoretical and technical limitations [46]. To address the disadvantages of nitrogen balance studies, the stable isotope-based indicator AA oxidation (IAAO) method has been proposed to derive IAA and protein requirements [52]. The IAAO method identifies protein requirements that maximize systemic protein synthesis [53], and generally suggests higher protein requirements (EAR of 0.87–0.93 g/kg BW/d for general young adults) [9]. However, it remains to be determined whether requirements should be based on compensation of losses, or maximal protein synthesis rates [52]. In the latter case, prevalence of inadequacy as estimated in this study would drastically increase.

In conclusion, this study shows how the PAQS can enhance the estimation of daily utilizable protein intake. Furthermore, this study estimates the prevalence of protein inadequacy by applying the EAR cut-point method, after accounting for energy misreporting, within-subject variation, and nonnormality of the intake distribution. Considering utilizable protein intake in 25 European countries, the prevalence of inadequate protein intake among females was <7%, except for Germany (17%). For males, the prevalence was below 6%. Before correcting for quality, the prevalence of inadequacy for all countries was nil, showing the difference that arises when adjusting for protein quality. These findings indicate that with the current diet composition and quantity of protein intake there is no cause for concern in the healthy adult European population, with a possible exception for females in Germany. The prevalence of protein inadequacy in sustainable, and thereby more plant-based diets, remains uncertain. Therefore, protein quality must be carefully considered when evaluating protein adequacy of these future diets.

## Author contributions

The authors’ responsibilities were as follows – SNH: conceptualization, data curation, formal analysis, visualization, investigation, software, methodology, and writing – original draft; SB: project administration, supervision, writing – review and editing, and funding acquisition; MCD: resources, writing – review and editing, AK: writing– review and editing, and supervision; MG: resources, data curation, and writing – review and editing; JMG: writing - review and editing and supervision; PvV: methodology, writing – review and editing, and supervision; and all authors: read and approved the final manuscript.

## Data availability statement

Food consumption data can be requested for at https://www.efsa.europa.eu/en/data-report/food-consumption-data (Accessed on 2 January, 2023). Dutch food composition data (NEVO-online version 2021/7.1) is available through https://nevo-online.rivm.nl/. Amino acid composition data can be requested for at dietetiek@wur.nl.

## Declaration of generative AI and AI-assisted technologies in the writing process

ChatGPT, an artificial intelligence language model developed by OpenAI, was consulted to refine the manuscript’s phrasing.

## Funding

This work was supported by a research grant provided by the VLAG Graduate School of Wageningen University, Wageningen, The Netherlands. This work received funding from the European Union’s Horizon 2020 research and innovation program under grant agreement number 101059632 (project GIANT LEAPS).

## Conflict of interest

The authors declare no conflict of interest.
