# Supplementary material for: Protein Adequacy in Europe: Adjusting Crude Intakes Using the Protein Adequacy and Quality Score (PAQS)
Source: Curr Dev Nutr. 2025 Aug 29;9(10):107539. doi: 10.1016/j.cdnut.2025.107539 (PMC12493195; doi:10.1016/j.cdnut.2025.107539)
Supplement: Multimedia component 1 [file mmc1.docx]

# Supplementary material for

# Protein adequacy in Europe: adjusting crude intakes using the Protein Adequacy and Quality Score (PAQS)

Samantha Nikita Heerschop^a^, Sander Biesbroek^a^, Merel Celine Daas^a^, Anneleen Kuijsten^a^, Mirjana Gurinović^b^, Johanna Marianna Geleijnse^a^, Pieter van ’t Veer^a^.

a: Division of Human Nutrition and Health, Wageningen University & Research, Wageningen, Gelderland, The Netherlands;

b: Centre of Research Excellence in Nutrition and Metabolism, Institute for Medical Research, National Institute of Republic of Serbia, University of Belgrade, Belgrade, Serbia. Capacity Development in Nutrition (CAPNUTRA), Belgrade, Serbia

**Corresponding author:**

Sander Biesbroek

sander.biesbroek@wur.nl

Postal address: Division of Human Nutrition and Health, P.O. 17, 6700 AA Wageningen, The Netherlands

**ORCIDs of the authors:**

Samantha Heerschop: 0000-0002-0091-7308

Sander Biesbroek: 0000-0001-9934-0969

Merel Daas: 0009-0004-7909-2434

Anneleen Kuijsten: 0000-0001-8120-7027

Mirjana Gurinović : [0000-0002-8649-6267](https://orcid.org/0000-0002-8649-6267)

Johanna M. Geleijnse: 0000-0001-7638-0589

Pieter van ’t Veer: 0000-0002-9708-3513

Key words: protein quality, utilizable protein, protein requirements, indispensable amino acids, PAQS, European adult population, EFSA EU Menu.

## Supplementary table 1

Supplemental table 1. Details of national food consumption surveys for the adult population available from the EFSA Comprehensive European Food Consumption Database.

| **Country** | **Survey name** | **Period** | **Method of dietary assessment** | **Number of days** | **Consecutive measurement days** | **Age range** | **Sample size** | **According to EFSA menu methodology (1)** |
| --- | --- | --- | --- | --- | --- | --- | --- | --- |
| Austria | AT-NATIONAL-2016 | 2014-2018 | 24-h dietary recall | 2 | No | 18-64 | 2250 | Yes |
| Belgium | NATIONAL-FCS-2014 | 2014-2015 | Food record, 24-h dietary recall | 2 | No | 3-64 | 3305 | Yes |
| Bosnia and Herzegovina | B&H MENU | 2017-2020 | 24-h dietary recall | 2 | No | 10-64 | 1529 | Yes |
| Bulgaria | NSFIN | 2004 | 24-h dietary recall | 1 | NA | 16-94 | 1204 | No |
| Croatia | NIPNOP-HAH-2011-2012 | 2011-2012 | 24-h dietary recall | 3 | No | 18-64 | 2002 | Yes |
| Cyprus | CY 2014-2017-LOT2 | 2014-2017 | 24-h dietary recall | 2 | No | 10-76 | 1016 | Yes |
| Czech Republic | SISP04 | 2003-2004 | 24-h dietary recall | 2 | No | 4-64 | 2353 | No |
| Denmark | DANSDA 2005-08 | 2005-2008 | Food record | 7 | Yes | 4-75 | 2700 | No |
| Estonia | DIET-2014-EST-A | 2013-2015 | 24-h dietary recall | 2 | No | 11-75 | 3049 | Yes |
| Finland | FINDIET 2017 | 2017 | 24-h dietary recall | 2 | No | 18-75 | 1773 | Yes |
| France | INCA3 | 2014-2015 | Food record, 24-h dietary recall | 3 | No | 0-79 | 4847 | Yes |
| Germany | NATIONAL NUTRITION SURVEY II | 2007 | 24-h dietary recall | 2 | No | 14-80 | 13,926 | No |
| Greece | GR-EFSA-LOT2 2014-2015 | 2014-2016 | 24-h dietary recall | 2 | No | 10-75 | 798 | Yes |
| Hungary | EU MENU DIETARY SURVEY OF HUNGARY | 2018-2020 | Food record, 24-h dietary recall | 2 | No | 1-74 | 2689 | Yes |
| Ireland | NANS 2012 | 2008-2010 | Food record | 4 | Yes | 18-90 | 1500 | No |
| Italy | IV SCAI ADULT 2018-2020 | 2018-2020 | 24-h dietary recall | 2 | No | 10-74 | 1203 | Yes |
| Latvia | LATVIA_2014 | 2012-2015 | Food record, 24-h dietary recall | 2 | No | 0-80 | 3595 | Yes |
| Montenegro | EUMENU ADLT | 2017-2021 | 24-h dietary recall | 2 | No | 10-74 | 1513 | Yes |
| Netherlands | FCS2016_CORE | 2012-2016 | Food record, 24-h dietary recall | 2 | No | 1-80 | 4313 | Yes |
| Poland | IZZ FAO 2000 | 2000 | 24-h dietary recall | 1 | NA | 1-95 | 4134 | No |
| Portugal | IAN-AF 2015-2016 | 2015-2016 | Food record, 24-h dietary recall | 2 | No | 0-84 | 6429 | Yes |
| Romania | RO-DIET-NATIONAL-STUDY-2019 | 2019-2020 | 24-h dietary recall | 2 | No | 10-74 | 1730 | Yes |
| Serbia | RS_ADULTS | 2019-2020 | 24-h dietary recall | 2 | No | 10-75 | 2737 | Yes |
| Slovakia | SK MON 2008 | 2008 | 24-h dietary recall | 1 | NA | 19-59 | 2761 | No |
| Slovenia | SI.MENU-2018 | 2017-2018 | Food record, 24-h dietary recall | 2 | No | 0-74 | 1981 | Yes |
| Spain | ENALIA2 | 2013-2015 | 24-h dietary recall | 2 | No | 18-74 | 968 | Yes |
| Sweden | RIKSMATEN 2010 | 2010-2011 | Web-based food record | 4 | Yes | 18-80 | 1797 | No |
| United Kingdom | NDNS ROLLING PROGRAMME YEARS 1-3 | 2008-2011 | Food record | 4 | Yes | 1-94 | 3073 | No |

NA: not applicable. 1. EFSA. (2014) Guidance on the EU Menu methodology. EFSA journal.12(12):3944. <https://doi.org/10.2903/j.efsa.2014.3944>.

## Supplementary table 2

Supplemental table 2. Number of measured consumption days for adults aged 18-64, per country, excluding pregnant and lactating women, and excluding participants in the lowest and highest 1.25% of reported energy intakes in each country.

|  | Number of measured consumption days per individual | | | | | | |
| --- | --- | --- | --- | --- | --- | --- | --- |
|  | **1** | **2** | **3** | **4** | **5** | **6** | **7** |
| Country |  |  |  |  |  |  |  |
| Austria | 76 | 2096 | 0 | 0 | 0 | 0 | 0 |
| Belgium | 63 | 1137 | 0 | 0 | 0 | 0 | 0 |
| Bosnia and Herzegovina | 5 | 826 | 0 | 0 | 0 | 0 | 0 |
| Bulgaria | 673 | 0 | 0 | 0 | 0 | 0 | 0 |
| Croatia | 0 | 0 | 1929 | 0 | 0 | 0 | 0 |
| Cyprus | 3 | 228 | 23 | 0 | 0 | 0 | 0 |
| Czech Republic | 0 | 1624 | 0 | 0 | 0 | 0 | 0 |
| Denmark | 0 | 0 | 0 | 3 | 10 | 12 | 1615 |
| Estonia | 58 | 1982 | 0 | 0 | 0 | 0 | 0 |
| Finland | 98 | 1147 | 0 | 0 | 0 | 0 | 0 |
| France | 195 | 99 | 1635 | 0 | 0 | 0 | 0 |
| Germany | 0 | 10071 | 0 | 0 | 0 | 0 | 0 |
| Greece | 1 | 251 | 0 | 0 | 0 | 0 | 0 |
| Hungary | 6 | 483 | 0 | 0 | 0 | 0 | 0 |
| Ireland | 0 | 0 | 0 | 1242 | 0 | 0 | 0 |
| Italy | 28 | 683 | 0 | 0 | 0 | 0 | 0 |
| Latvia | 123 | 1061 | 0 | 0 | 0 | 0 | 0 |
| Montenegro | 10 | 679 | 0 | 0 | 0 | 0 | 0 |
| Netherlands | 0 | 1440 | 0 | 0 | 0 | 0 | 0 |
| Poland | 2417 | 0 | 0 | 0 | 0 | 0 | 0 |
| Portugal | 285 | 2924 | 0 | 0 | 0 | 0 | 0 |
| Romania | 113 | 718 | 0 | 0 | 0 | 0 | 0 |
| Serbia | 5 | 1113 | 0 | 0 | 0 | 0 | 0 |
| Slovakia | 2686 | 0 | 0 | 0 | 0 | 0 | 0 |
| Slovenia | 7 | 362 | 0 | 0 | 0 | 0 | 0 |
| Spain | 14 | 522 | 0 | 0 | 0 | 0 | 0 |
| Sweden | 0 | 15 | 8 | 1282 | 0 | 0 | 0 |
| United Kingdom | 0 | 0 | 23 | 1209 | 0 | 0 | 0 |

## Supplementary methods 1

**Explanation of derivation of the Protein Adequacy and Quality Score (PAQS)**

Authors: Pieter van ’t Veer, Samantha N. Heerschop

Version: 1.0

Definition of the PAQS

This file describes the Protein Adequacy and Quality Score (PAQS). The PAQS is a measure of protein adequacy **for the diet as a whole**, in which the amount of ingested protein is corrected for protein quality **of each meal**. The PAQS compares utilizable protein intake with the protein requirement. Current methods such as the Protein Digestibility-Corrected Amino Acid Score (PDCAAS) and Digestible Indispensable Amino Acid Score (DIAAS) account for protein quality of single foods or meals, whereas the evaluation of protein adequacy of whole diets currently accounts for the quantity of crude protein intake. The PAQS combines quality and quantity of dietary protein into a single measure and can therefore be used to evaluate protein adequacy of the diet as a whole. As the current protein requirements are defined in terms of intakes required to meet metabolic needs for maintenance, utilizable protein intake rather than crude protein intake should be used to assess protein adequacy. Besides, the current protein requirement is expressed per day, therefore utilizable protein should also be calculated over the day. A more detailed overview of protein quality scoring methods is provided in Supplemental table 4.

We define the PAQS as shown in equation *(1)*:

| $PAQS=\frac{Daily utilizable protein intake}{Daily protein requirement}\geq1 implies protein adequacy$ | $\left[ 1 \right]$ |
| --- | --- |

To calculate the PAQS, data is needed on the daily intake of foods and amino acids (AAs) at the level of meals. In addition, to assess utilizable protein, literature data is needed on protein digestion and absorption, and requirements of specific amino acids (AAs).

Physiological background

Below, some definitions regarding the physiological background for applying the PAQS are explained.

- Utilized AAs: utilized AAs are those AAs from the diet that are used for protein synthesis and incorporated into body proteins. This is an unknown quantity.
- Utilizable AAs: Utilizable AAs are those AAs that are bioavailable for body protein synthesis but may eventually not be incorporated. In other words, these AAs appear in the bloodstream after consumption (= intake), digestion, and absorption in a form that can be utilized for body protein synthesis and other pathways which constitute the metabolic demand (1). The profile of utilizable AAs corresponds with the profile that is needed for protein synthesis.
- Absorbed indispensable amino acids (IAAs) and dispensable amino acids (DAAs): AAs after digestion and absorption has taken place. The profile of absorbed AAs differs from intake because of digestion but does not necessarily correspond to the profile that is needed for protein synthesis, as opposite to utilizable AAs.
- Meals: Amino acids of different products can complement each other when they are simultaneously present in the bloodstream (2). The exact time window in which amino acids can complement each other is not yet clear. The PAQS assumes that complementary AAs should be available within the relatively short time window after a meal, and the amount of utilizable AAs should be calculated per meal as well. We adopted the following definition for a meal: the food served and eaten especially at one of the customary, regular occasions for taking food during the day, as breakfast, lunch, or supper (3). Snacks in between meals are also considered as separate meals and thus contribute to the daily utilizable protein intake.
- Note that the basic assumption is that the profile of required amino acids according to the FAO (Supplemental table 3) corresponds to the requirement profile for a meal. In an example calculation FAO applies their protein quality score (DIAAS) on a meal, indicating it is intended that way (1).
- When an IAA exceeds its requirement, it can be metabolized to DAAs. In that case, the surplus IAA is not utilized for protein synthesis and – after metabolic conversion – it can be used as DAA instead. Assuming that this conversion of the surplus of IAAs into DAAs functions well (i.e. 100% conversion), DAAs will not become limiting, and they do not affect protein adequacy. However, when the conversion is diminished, DAAs may become limiting. Current methods do not consider DAAs as potential limiting amino acid. Therefore, the PAQS includes the requirement for DAAs in case the conversion of IAAs into DAAs would be limited.

Amino acid requirements

In this document, requirements of AAs (in mg/kg BW/d) refer to Estimated Average Requirements (EARs) needed for protein synthesis, as provided by Table 5 the Food and Agriculture Organization of the United Nations (1) (Supplemental table 3).

Supplemental table 3. Amino acids needed for protein synthesis in the human body of adults, expressed by two related metrics, based on Table 5 from Food and Agriculture Organization of the United Nations (1).

| Metric | IAAs | | | | | | | | | DAAs | Protein |
| --- | --- | --- | --- | --- | --- | --- | --- | --- | --- | --- | --- |
|  | His | Ile | Leu | Lys | SAA | AAA | Thr | Trp | Val |  |  |
| **Requirements** |  |  |  |  |  |  |  |  |  |  |  |
| mg/kg BW/d | 11 | 20 | 40 | 32 | 15 | 27 | 17 | 4.4 | 26 | 468 | 660 |
| parameter | $R_{b}$ | | | | | | | | | *RDAA* | *PR* |
| **Reference pattern** |  | | | | | | | | |  |  |
| mg/g protein | 16 | 30 | 61 | 48 | 23 | 41 | 25 | 6.6 | 40 | 709 | 1000 |
| parameter | ${R1g}_{b}$ | | | | | | | | | *RDAA1g* |  |

His, histidine; Ile, isoleucine; Leu, leucine; SAA, sulphur amino acids; AAA, aromatic amino acids, Thr, threonine, Trp, tryptophan; Val, valine. BW, body weight; $R_{b}$, Requirement of indispensable amino acid *b* in mg/kg BW/d. ${R1g}_{b}$, Reference pattern of indispensable amino acid *b* in mg/g protein. *RDAA*, requirement of dispensable amino acids in mg/kg BW/d. *RDAA1g*, reference pattern of dispensable amino acids in mg/g protein. *PR*, protein requirement in g/kg BW/d.

In the PAQS, these requirements can be adjusted according to the needs of specific population groups if needed. Also, if the PAQS is used for diet planning of an individual, the protein and amino acid requirements in Supplemental table 3 can be substituted by the corresponding Recommended Daily Allowances (RDAs).

Indices and parameters

Below, some indices and parameters are explained.

**Indices**

*b* index for indispensable amino acids (IAAs).

*c* index for dispensable amino acids (DAAs).

*j*  index for food item, i.e. potato, cheese, etc.

*m* index for meal moment, i.e. breakfast, lunch, dinner.

**Parameters related to the diet**

*F_jm_* Consumed food item *j* during meal moment *m* in g.

*W* Body weight (BW) of individual in kg.

*CIAA_bj_* Composition of indispensable amino acid *b* of food item *j* in mg/100g.

*CDAA_cj_* Composition of dispensable amino acid *c* of food item *j* in mg/100g.

*IIAA_bjm_* Intake of indispensable amino acid *b* from food item *j* from meal moment *m* in mg/kg BW.

*IDAA_cjm_* Intake of dispensable amino acid *c* from food item *j* from meal moment *m* in mg/kg BW.

*ITAA_m_* Intake of total amount of amino acids in meal moment *m* in mg/kg BW, i.e. the sum of all IAA and DAA.

**Parameters related to absorption**

*TID_bj_* True Ileal Digestibility (TID) of indispensable amino acid *b* in food item *j* in fraction.

*TID_cj_* True Ileal Digestibility (TID) of dispensable amino acid *c* in food item *j* in fraction.

*AIAA_bjm_* Absorbed indispensable amino acid *b* from food item *j* from meal moment *m* in mg/kg BW.

*AIAA1g_bj_* Pattern of absorbed indispensable amino acid *b* from food item *j* in mg/g protein.

*ADAA_cjm_* Absorbed dispensable amino acid *c* from food item *j* from meal moment *m* in mg/kg BW.

*ADAA1g_cj_* Pattern of absorbed dispensable amino acid *c* from food item *j* in mg/g protein.

*ATAA_m_* Absorbed total amount of amino acids in meal moment *m* in mg/kg BW, i.e. the sum of all absorbed IAA and DAA.

**Parameters related to requirements**

*R_b_*  Requirement of indispensable amino acid *b* in mg/kg BW/d (Supplemental table 3).

*R1g_b_*  Requirement pattern of indispensable amino acid *b* in mg/g protein (Supplemental table 3).

*RDAA* Requirement of dispensable amino acids in mg/kg BW/d.

*RDAA1g* Requirement pattern of dispensable amino acids in mg/g protein.

*PR*  Protein requirement in g/kg BW/d.

Amino acid intake

Equation *(2)* shows the intake of indispensable amino acid *b* from food item *j* from meal moment *m* (${IIAA}_{bjm}$) in mg/kg BW. Equation *(3)* shows the intake of dispensable amino acid *c* from food item *j* from meal moment *m* (${IDAA}_{cjm}$) in mg/kg BW.

| ${IIAA}_{bjm}=\frac{F_{jm}}{W}*\frac{{CIAA}_{b}}{100} \forall bjm$ | $\left[ 2 \right]$ |
| --- | --- |
| ${IDAA}_{cjm}=\frac{F_{jm}}{W}*\frac{{CDAA}_{c}}{100} \forall cjm$ | [3] |

Where $F_{jm}$ is the consumed amount of food item *j* during meal moment *m* in g. $W$ is the body weight of an individual in kg. ${CIAA}_{b}$ is the amount of indispensable amino acid *b* of food item *j* in mg/100g. ${CDAA}_{c}$ is the amount of dispensable amino acid *c* of food item *j* in mg/100g.

Equation *(4)* shows the total intake of indispensable and dispensable amino acids in meal moment *m* in mg/kg BW (${ITAA}_{m}$) as the sum of ${IIAA}_{bjm}$ and ${IDAA}_{cjm}$.

| ${ITAA}_{m}= \sum_{bj} {IIAA}_{bjm}+\sum_{cj} {IDAA}_{cjm}\forall m$ | [4] |
| --- | --- |

Amino acid digestibility

After intake, amino acid digestion and absorption take place. Amino acids are consumed in meals. Products *j* within a meal may have different digestibility factors, or even the indispensable amino acids *b* and dispensable amino acids *c* within a product *j* may have different digestibility factors. To move from intake $IIAA$ of indispensable amino acid *b* from food item *j* from meal moment *m* to absorbed $AIAA$ indispensable amino acid *b* from food item *j* from meal moment *m*, intake should be multiplied by a digestibility value of amino acid *b* of food item *j*, as shown in equation *(5)*.

| ${AIAA}_{bjm}={TID}_{bj}*{IIAA}_{bjm} \forall bjm$ | $\left[ 5 \right]$ |
| --- | --- |
| ${ADAA}_{cjm}={TID}_{cj}*{IDAA}_{cjm} \forall cjm$ | $\left[ 6 \right]$ |

Where ${TID}_{bj}$ and ${TID}_{cj}$ are the True Ileal Digestibility of respectively amino acids *b* and *c* in food item *j*. The total of all absorbed amino acids $ATAA$ *b* and *c* of all products *j* in meal moment *m* (in mg/kg BW) is calculated in equation *(7)*.

| ${ATAA}_{m}= \sum_{bj} {AIAA}_{bjm}+\sum_{cj} {ADAA}_{cjm}\forall m$ | $\left[ 7 \right]$ |
| --- | --- |

Calculation of the PAQS

Protein adequacy that accounts for protein quality of meals relies on a combination of three conditions that must be met:

(1) requirements for the IAAs according to the reference profile,

(2) requirements for the DAAs, i.e. the appropriate ratio of IAAs and DAAs and

(3) meeting the daily protein (Nitrogen) requirement

The first two components relate to protein quality and are derived from protein digestibility and amino acid (AA) reference profiles (Supplemental table 3). Therefore, (1) and (2) should be calculated on a meal basis.

Equation *(1a)* expresses all components (1), (2), and (3).

| $PAQS=\sum_{m} \left( MIN\left( \frac{\sum_{j} {AIAA}_{bjm}}{R_{b}},\frac{\sum_{cj} {ADAA}_{cjm}}{RDAA},\frac{{ATAA}_{m}}{PR} \right) \right)$ | $\left[ 1a \right]$ |
| --- | --- |

Where $R_{b}$ is the required amount of utilizable IAAs *b* in mg/kg BW/d, $RDAA$ is the required amount of utilizable DAAs in mg/kg BW/day, $PR$ is the total required amount of utilizable IAAs and DAAs in g/kg BW/day (equals EAR = 0.66 g/kg BW/d for adults according to the Food and Agriculture Organization of the United Nations (1)).

Equation *(1a)* takes the lowest of the three conditions for each meal, and sums over the meals to obtain the PAQS.

Application of the PAQS

To obtain the percentage of the population with inadequate protein intake according to the PAQS, methods like the NCI method or SPADE can be applied to correct for day-to-day variability within subjects by using PAQS = 1 as the requirement (4-6). These methods estimate usual intake distributions and subsequently use the EAR cut-point method to define the adequacy of a population.

Equation *(1a)* is a linear formula and can thus be applied as a constraint or an objective function in diet optimization studies that use linear programming.

Relation of PAQS with other protein quality measures

This section elaborates on the relation of the PAQS with other protein quality measures such as the DIAAS, the PDCAAS, and the EAA-9, and provide an overview of the primary aim and main features of five protein quality scoring methods, compared to the PAQS (Supplemental table 4), and the data input required for each protein quality scoring method (Supplemental table 5).

*DIAAS*

According to the FAO, the DIAAS is the preferred measure to quantify protein quality (equation *(8)*) (1).

| $DIAAS\%=100*\frac{mg of digestible dietary IAA in 1g of dietary protein}{mg of the same dietary IAA in 1g of the reference protein}$ | $\left[ 8 \right]$ |
| --- | --- |

According to the FAO, equation *(8)* should be calculated for each dietary IAA. The lowest value of DIAAS% of the 1-9 IAAs is designated as the DIAAS and used as an indicator of dietary protein quality.

In the following part we will show how the PAQS relates to the DIAAS. For this equation *(1a)* needs to be rewritten by factoring out $\left( \frac{ATAA_{m}}{PR} \right)$ of the minimum (forward). Equation *(1b)* shows the intermediate steps, equation *(1c)* shows the result of the factoring out.

| $PAQS=\sum_{m} \left( \frac{ATAA_{m}}{PR} \right)MIN\left( \frac{\left( \frac{\sum_{j} {AIAA}_{bjm}}{ATAA_{m}} \right)}{\left( \frac{R_{b}}{PR} \right)},\frac{\left( \frac{\sum_{cj} {ADAA}_{cjm}}{ATAA_{m}} \right)}{\left( \frac{RDAA}{PR} \right)},\frac{\left( \frac{ATAA_{m}}{ATAA_{m}} \right)}{\left( \frac{PR}{PR} \right)} \right)$ | $\left[ 1b \right]$ |
| --- | --- |
| $PAQS=\sum_{m} \left( \frac{ATAA_{m}}{PR} \right)MIN\left( \frac{\sum_{j} {AIAA1g}_{bjm}}{{R1g}_{b}},\frac{\sum_{j} ADAA1g_{cjm}}{RDAA1g},1 \right)$ | $[1c]$ |

Where ${AIAA1g}_{bj}$ is the absorbed indispensable amino acid *b* from food item *j* from meal moment *m* in mg/g protein. ${R1g}_{b}$ is the requirement of indispensable amino acid *b* in mg/g protein. $ADAA1g_{cj}$ is the absorbed dispensable amino acid *c* from food item *j* from meal moment *m* in mg/g protein. $RDAA1g$ is the requirement of dispensable amino acids in mg/g protein.

In equation *(1c)* all terms within the MIN(..) are dimensionless amino acid profiles (mg or g per g protein); the match of the profiles is expressed as the ratios of the percentage of the absorbed amino acids (numerators) and the percentage of required amino (denominator). The definition of the DIAAS as shown in equation (8) corresponds to the green part of the MIN(..) in equation *(1c)*. Furthermore, equation *(1c)* shows that factoring out $\left( \frac{ATAA_{m}}{PR} \right)$ from *(1a)* introduces truncation of the MIN at 1 when AAs are expressed as profiles (in mg/g protein). Note that $ATAA_{m}$ is derived from TIDs that are specific for each amino acid (equation 5 to 7).

*PDCAAS*

Before the DIAAS was introduced, the PDCAAS was used as a measure of protein quality. Equation *(9)* shows the definition of PDCAAS by the WHO, FAO, and UNU (7).

| $PDCAAS=digestibility*\frac{mg of amino acid in 1g test protein}{mg of amino acid in requirement protein}$ | $\left[ 9 \right]$ |
| --- | --- |

In case the digestibility ${TID}_{bj}$ in the DIAAS is similar for all IAAs *b*, the subscript *b* cancels from equation *(5).* Therefore, the PDCAAS is a special case of the DIAAS*,* the difference being that the DIAAS applies an amino acid-specific digestibility for each food, while for PDCAAS it is assumed that all IAAs in the same food have a similar digestibility. Furthermore, the DIAAS uses TID, while in PDCAAS faecal digestibility is used. The PAQS allows to use either way, depending on the availability of the data.

In the theoretical case that the TID of all individual AAs would be identical for one food, the overall protein digestibility of that food would correspond to its true faecal digestibility, i.e. the faecal digestibility corrected for intestinal protein losses on a protein free diet. However, empirical data on either ileal digestibility or true digestibility are often derived from different clinical patient groups and/or animal or in vitro experiments which might add systematic differences between these estimates and limit their comparability.

*EAA-9*

The mathematical framework of the Essential Amino Acid 9 (EAA-9) scoring system is as follows (8):

| $EAA-9=MIN(\frac{His \left( \frac{mg}{svg} \right)}{His RDA}, \frac{Ile \left( \frac{mg}{svg} \right)}{Ile RDA},\frac{Leu \left( \frac{mg}{svg} \right)}{Leu RDA},\frac{Lys \left( \frac{mg}{svg} \right)}{Lys RDA},$  $\frac{Met+Cys \left( \frac{mg}{svg} \right)}{Met+Cys RDA},\frac{Phe+Tyr \left( \frac{mg}{svg} \right)}{Phe+Tyr RDA},\frac{Thr \left( \frac{mg}{svg} \right)}{Thr RDA},$  $\frac{Trp \left( mg/svg \right)}{Trp RDA},\frac{Val \left( mg/svg \right)}{Val RDA})*100$ | $\left[ 10 \right]$ |
| --- | --- |

*Where svg is serving. RDAs are defined in mg/70 kg BW/d, converted to mg/g of protein by dividing by RDA for protein (0.8 g/kg BW/d = 56 g/70 kg BW/d). FAO/WHO/UNU scoring pattern defined in mg/g protein converted into mg/70 kg BW/d by multiplying by the FAO mean protein recommendation (0.66 g/kg BW/d = 42.6 g/70 kg BW/d), cited from Forester, Jennings-Dobbs (8).*

The EAA-9 corresponds to the to $\frac{\sum_{j} {AIAA}_{bjm}}{R_{b}}$ of the MIN(..) in equation *(1a)*, when the amino acid values in the EAA-9 are expressed per kg BW and to $\frac{\sum_{j} {AIAA1g}_{bjm}}{{R1g}_{b}}$ of the MIN(..) in equation *(1c)*, when they are expressed per g protein. The EAA-9 accounts for protein quantity by multiplying the RDA of an IAA by the protein requirement of 0.8 g/kg BW/d and by BW, whereas the PAQS expresses intakes per kg BW, and accounts for protein quantity by the red part of equation *(1a)* and *(1c)*. Note that the RDA is a measure to plan individual nutrient intakes, whereas the EAR, as used in the PAQS, is a measure to evaluate population nutrient intakes (9). Obviously, the EAR in the PAQS can be replaced by the RDA in case an individual planning needs to be made.

The EAA-9 can be applied additively or cumulatively. With the cumulative method, the EAA content of each food is summed prior to scoring (EAA-9_Cumulative_ = EAA-9_(Food A + Food B)_), which allows amino acids form different foods to complement each other. This corresponds to the way how the PAQS calculates utilizable protein for a meal. With the additive method, each food is scored individually, and the resulting scores are added (EAA-9_Additive_ = EAA-9_Food A_ + EAA-9_Food B_), i.e. the amino acids from these foods cannot complement each other. This is analogous to the addition of the PAQS over meals.

*MPQS*

The Meal Protein Quality Score (MPQS) is a tool that integrates digestibility-adjusted IAA intake with total protein consumed in a meal, which, together with the IAA requirements that correspond to the total protein requirement of 0.3 g/kg body weight, provides a score from 0 to 100 to reflect IAA coverage adequacy **for a meal**. Because of the total protein requirement of 0.3 g/kg body weight, the MPQS focusses on optimizing protein intake for vulnerable populations, particularly older adults. Assuming consumption of three main meals and snacks, total protein intake will be around 1.0–1.2 g/kg/d, which is in line with official recommendations for older adults (10). The formula of the MPQS is shown in equation *(11)*.

| $MPQS=MIN\left( \% \right)_{i}\left( \frac{\sum_{j} (intake \left( mg \right) of EAA_{i} from food_{j} x digestibility factor of food_{j}}{personalised requirement \left( mg \right) of EAA_{i}} \right)$ | $\left[ 11 \right]$ |
| --- | --- |

*Where i is an index for essential amino acid (EAA), also referred to as indispensable amino acid, and j is an index for food item.*

The MPQS corresponds to the $\frac{\sum_{j} {AIAA}_{bjm}}{R_{b}}$ of the MIN(..) in equation *(1a)*, but the MPQS expresses both the numerator and denominator as absolute amounts, whereas in the PAQS the numerator and denominator are expressed per kg body weight. This way the MPQS directly accounts for the quantity consumed, and this quantity has to be met within a meal. In the PAQS the third ratio of the MIN(..), i.e. $\frac{{ATAA}_{m}}{PR}$ accounts for the total quantity consumed. In the PAQS, the total protein requirement (0.66 g/kg/d) does not have to be met within one meal. One big meal can compensate for another smaller meal.

Summarized, there are four main differences between the PAQS and the MPQS: 1) the PAQS uses **daily** protein requirements (0.66g/kg body weight/day) for healthy adults, whereas the MPQS uses **meal** protein requirements (0.3 g/kg body weight) for older adults, 2) the MPQS allows for guidance in meal planning, whereas the PAQS allows for evaluation of population protein adequacy, 3) both the PAQS and the MPQS strictly account for the timeframe within which IAAs can complement each other, but the MPQS additionally establishes protein requirements per specific time interval, 4) in the PAQS it is possible to correct for impaired conversion of IAAs to DAAs.

Supplemental table 4. Primary aim and main features of four protein quality scoring methods, compared to the PAQS. Adapted from (8).

|  | DIAAS – Digestible Indispensable Amino Acid Score | PDCAAS – Protein Digestibility-Corrected Amino Acid Score | EAA-9 – Essential Amino Acid 9 score | MPQS – Meal Protein Quality Score | PAQS – Protein Adequacy and Quality Score |
| --- | --- | --- | --- | --- | --- |
| Primary aim of scoring method | Evaluate protein quality of foods or meals by accounting for ileal digestibility and the reference profile of IAAs. | Evaluate protein quality of foods or meals by accounting for faecal digestibility and the reference profile of IAAs. | Design personalized meals with personalized requirements | Assess protein quality and quantity of meals based on essential amino acid (EAA) content, digestibility, and requirements | Assess adequacy of whole diets while accounting for complementarity of AA profiles within meals * |
| Features of scoring method |  |  |  |  |  |
| Compare protein quality between foods | X | X | X |  |  |
| Can be personalized to individual needs, e.g. because of age or illness. |  |  | X | X | X |
| Either EARs or RDAs can be used to quantify requirements |  |  | X | X | X |
| Score of 100% guarantees that all 9 IAA requirements are met |  |  | X | X | X |
| Can design meals that meet the reference profile without complementarity of IAAs from different food items** |  |  | X |  |  |
| Can account for incomplete conversion of IAAs to DAAs |  |  |  |  | X |

* According to the terminology of Forester, Jennings-Dobbs (8), the PAQS is cumulative for food items within meals, and additive for different meals over the day.

** Usually, indispensable amino acids (IAAs) from different food items within a meal can complement each other towards complete IAA profiles. However, in some cases one may want to design meals including food items of which the IAAs are (theoretically) not able to complement each other.

Supplemental table 5. Data input required for each protein quality scoring method.

| Data input | FAO/WHO/ UNU AAS – amino acid score | DIAAS – Digestible Indispensable Amino Acid Score | PDCAAS – Protein Digestibility-Corrected Amino Acid Score | EAA-9 – Essential Amino Acid 9 score | MPQS – Meal Protein Quality Score | PAQS – Protein Adequacy and Quality Score |
| --- | --- | --- | --- | --- | --- | --- |
| IAA reference profile | X | X | X | X | X | X |
| Protein requirements |  |  |  |  |  | X |
| Amino acid composition data of foods | X | X | X | X | X | X |
| Digestibility data of individual AAs from foods |  | X |  | X | X | X |
| Digestibility data of whole proteins from foods |  |  | X |  | X* | X* |
| Food consumption data on meal level |  |  |  |  | X** | X |

*In case digestibility data of individual AAs from foods is not available, digestibility data of whole protein from foods can be used, as in PDCAAS.

** Food consumption data on meal level is optional for the MPQS. The tool is mainly focused on meal planning, for which only food composition data is required, but it can also be used to evaluate a consumed meal.

References

1. Food and Agriculture Organization of the United Nations. (2011) Dietary protein quality evaluation in human nutrition: Report of an FAO Expert Consultation. Auckland, New Zealand. Report No.: 0254-4725.

2. Adhikari S, Schop M, de Boer IJM, Huppertz T. (2022) Protein Quality in Perspective: A Review of Protein Quality Metrics and Their Applications. Nutrients.14(5):947. <https://doi.org/10.3390/nu14050947>

3. Dictionary.com. Meal [Available from: https://www.dictionary.com/browse/meal.

4. Dekkers AL, Verkaik-Kloosterman J, van Rossum CT, Ocké MC. (2014) SPADE, a new statistical program to estimate habitual dietary intake from multiple food sources and dietary supplements. The Journal of nutrition.144(12):2083-91. <https://doi.org/10.3945/jn.114.191288>

5. Dodd KW, Guenther PM, Freedman LS, Subar AF, Kipnis V, Midthune D, Tooze JA, et al. (2006) Statistical methods for estimating usual intake of nutrients and foods: a review of the theory. Journal of the American Dietetic Association.106(10):1640-50. <https://doi.org/10.1016/j.jada.2006.07.011>

6. Tooze JA, Kipnis V, Buckman DW, Carroll RJ, Freedman LS, Guenther PM, Krebs‐Smith SM, et al. (2010) A mixed‐effects model approach for estimating the distribution of usual intake of nutrients: the NCI method. Statistics in medicine.29(27):2857-68. <https://doi.org/10.1002/sim.4063>

7. World Health Organization. (2007) Protein and amino acid requirements in human nutrition. World health organization technical report series(935):1.

8. Forester SM, Jennings-Dobbs EM, Sathar SA, Layman DK. (2023) Perspective: Developing a Nutrient-Based Framework for Protein Quality. The Journal of Nutrition. <https://doi.org/10.1016/j.tjnut.2023.06.004>

9. Institute of Medicine. Dietary Reference Intakes: Applications in Dietary Assessment. Washington, DC: The National Academies Press; 2000.

10. Grootswagers P, Christensen SH, Timmer M, Riley W, de Groot L, Tetens I. (2024) Meal Protein Quality Score: A novel tool to evaluate protein quantity and quality of meals. Current Developments in Nutrition:104439. <https://doi.org/10.1016/j.cdnut.2024.104439>

## Supplementary figure 1


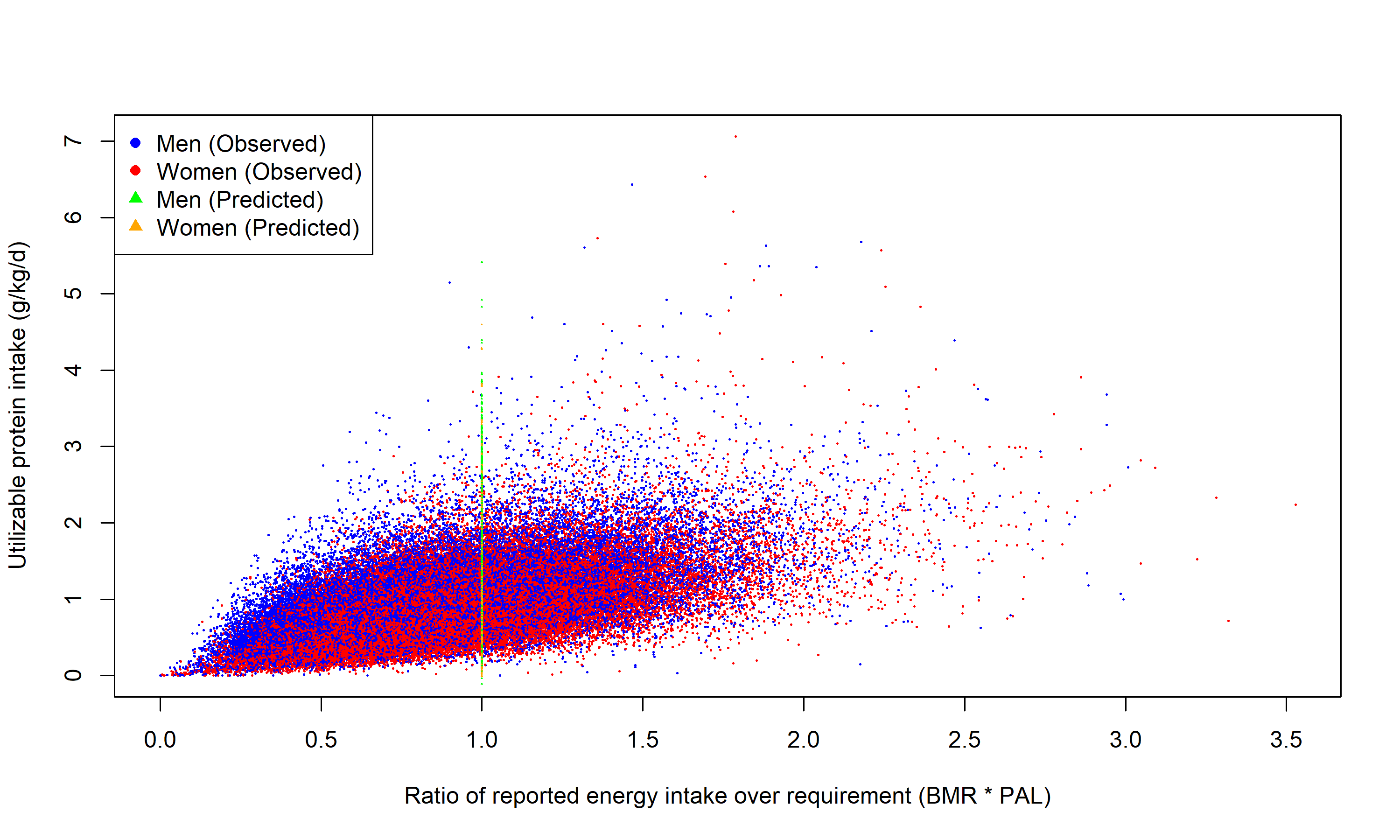


Supplemental figure 1. Scatter plot of observed and predicted utilizable protein intake (y-axis) and the ratio of energy intake over energy requirement (x-axis), by sex, derived from multiple 24-h dietary recalls or food records available in the harmonized European Food Safety Authority Comprehensive European Food Consumption Database. Daily utilizable protein intake was calculated using the Protein Adequacy and Quality Score (PAQS), by taking the sum of utilizable protein intake per meal considering amino acid digestibility, amino acid requirements, and crude protein intake. Energy requirement was defined as the basal metabolic rate, according to the Schofield equation, multiplied by a physical activity level (PAL) of 1.4. Utilizable protein intake was predicted by a linear mixed model, with protein intake (g/kg/d) as dependent variable, and the ratio of reported energy intake over required energy intake as independent variable. The model included a random intercept and random slope for subjects and adjusted for sex, country, and body mass index. An exponential covariance structure was applied to correctly consider consecutive and non-consecutive measurement days. Protein intake was predicted from this model at energy balance, i.e. for the ratio of reported energy intake over required energy intake equal to 1, assuming a PAL of 1.4.

## Supplementary figure 2


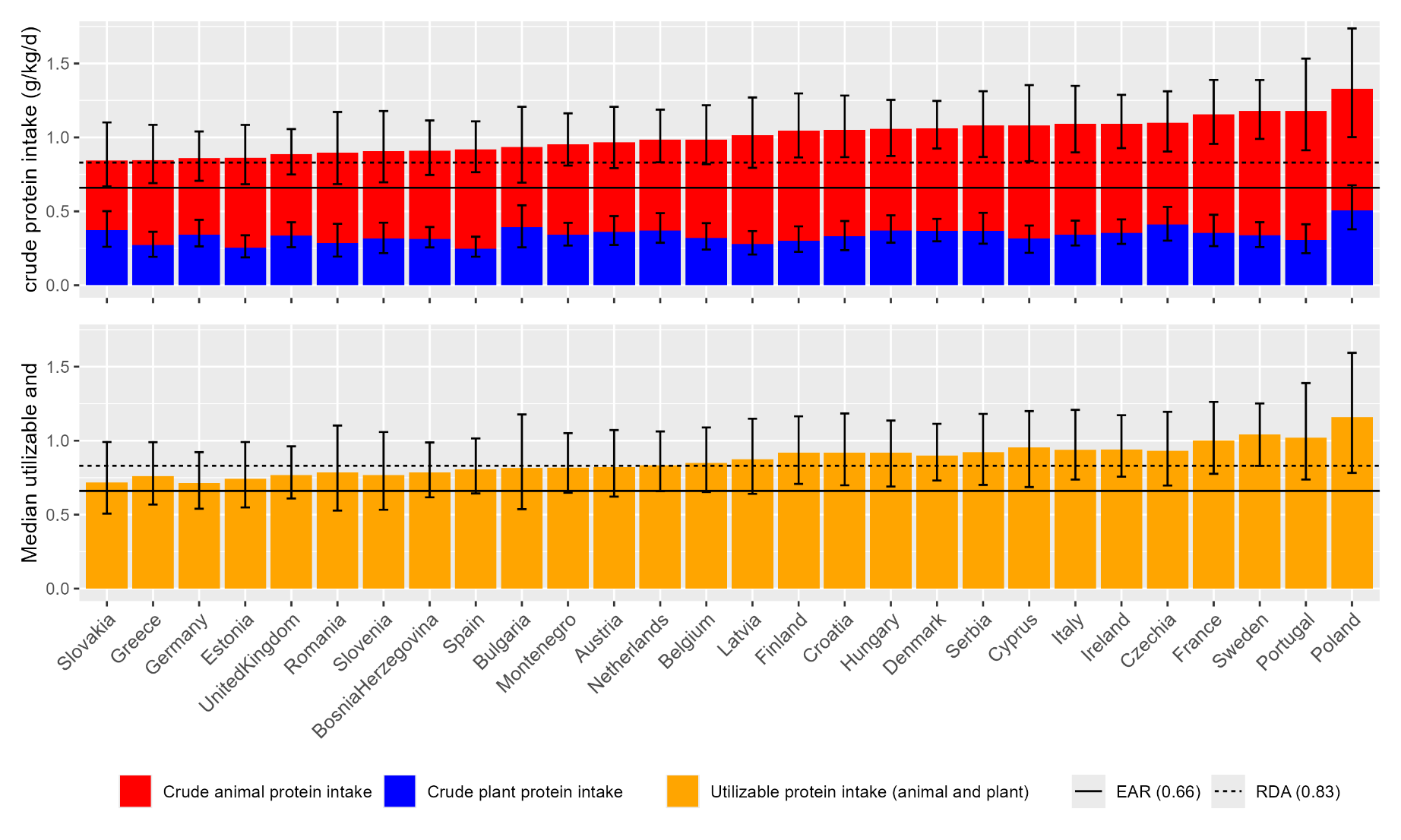


Supplemental figure 2. Median crude animal and plant protein intake (top) and utilizable protein intake (bottom) per European country in g/kg body weight/d, derived from multiple 24-h dietary recalls or food records available in the harmonized European Food Safety Authority (EFSA) Comprehensive European Food Consumption Database. Daily utilizable protein intake was calculated using the Protein Adequacy and Quality Score (PAQS), by taking the sum of utilizable protein intake per meal considering amino acid digestibility, amino acid requirements, and crude protein intake. Once utilizable protein intake is calculated using the PAQS, plant and animal protein can no longer be separated, as absorbed amino acids from different products within a meal are summed up and compared to the reference profile. EAR, Estimated Average Requirement. RDA: Recommended Dietary Allowance.

## Supplementary table 6

Supplemental table 6. Prevalence (%) of inadequate protein intake among adults (age 18 to 64) per European country stratified by sex, using either crude or utilizable protein intake, **based on predicted protein intake which is adjusted for energy misreporting,** or **on observed intake data including substantial percentages of under-reporting**. Data was derived from multiple 24-h dietary recalls or food records available in the harmonized European Food Safety Authority (EFSA) Comprehensive European Food Consumption Database. Daily utilizable protein intake was calculated using the Protein Adequacy and Quality Score (PAQS), by taking the sum of utilizable protein intake per meal considering amino acid digestibility, amino acid requirements, and crude protein intake. Prevalences were estimated by the Statistical Program to Assess usual Dietary Exposure (SPADE), which models usual intake distributions and removes within-person variation, and consequently applies the EAR cut-point method. Estimated Average Requirement (EAR) = 0.66 g/kg body weight/d.

| Country | Prevalence of inadequate **predicted**^1^ crude protein intake | | | Prevalence of inadequate **observed**^2^ crude protein intake | | | Prevalence of inadequate **predicted**^1^ utilizable protein intake | | | Prevalence of inadequate **observed**^2^ utilizable protein intake | | |
| --- | --- | --- | --- | --- | --- | --- | --- | --- | --- | --- | --- | --- |
|  | Total | Women | Men | Total | Women | Men | Total | Women | Men | Total | Women | Men |
| Austria | 0.2 (0.0-0.3) | 0.2 (0.0-0.4) | 0.1 (0.0-0.1) | 4.2 (2.8-5.5) | 5.1 (3.2-7.0) | 2.4 (0.8-4.1) | 5.9 (4.2-7.6) | 7.4 (4.9-9.9) | 3.1 (1.8-4.6) | 17.5 (14.6-20.2) | 21.1 (17.1-24.6) | 11.2 (7.5-14.8) |
| Belgium | 0.0 (0.0-0.0) | 0.0 (0.0-0.1) | 0.0 (0.0-0.0) | 3.8 (2.7-5.4) | 5.1 (3.1-8.4) | 2.5 (0.9-4.0) | 1.1 (0.4-1.9) | 1.7 (0.3-3.4) | 0.4 (0.0-0.9) | 13.2 (10.2-16.4) | 17.5 (12.6-22.9) | 8.7 (5.3-12.4) |
| Bosnia Herzegovina | 0.0 (0.0-0.0) | 0.0 (0.0-0.0) | 0.0 (0.0-0.0) | 6.5 (4.2-8.8) | 7.8 (4.3-10.9) | 5.2 (2.3-8.2) | 0.6 (0.1-1.3) | 1.0 (0.0-2.1) | 0.3 (0.0-0.8) | 20.2 (16.4-24.0) | 23.7 (17.8-29.0) | 16.5 (11.4-21.8) |
| Croatia | 0.0 (0.0-0.0) | 0.0 (0.0-0.0) | 0.0 (0.0-0.0) | 4.9 (3.8-6.1) | 6.7 (4.8-8.6) | 3.1 (1.8-4.5) | 0.4 (0.1-0.7) | 0.7 (0.2-1.2) | 0.1 (0.0-0.3) | 12.7 (10.6-14.6) | 16.9 (13.8-19.8) | 8.4 (5.8-10.8) |
| Cyprus | 0.0 (0.0-0.0) | 0.0 (0.0-0.0) | 0.0 (0.0-0.0) | 4.3 (1.4-7.3) | 8.2 (2.5-13.5) | 0.3 (0.0-1.9) | 0.3 (0.0-1.0) | 0.5 (0.0-1.6) | 0.1 (0.0-0.7) | 10.7 (6.9-15.5) | 19.4 (12.9-26.3) | 1.6 (0.0-7.4) |
| Czech Republic | 0.0 (0.0-0.0) | 0.0 (0.0-0.1) | 0.0 (0.0-0.0) | 4.4 (3.0-6.0) | 7.2 (4.6-10.0) | 1.4 (0.5-2.4) | 1.2 (0.1-2.4) | 2.1 (0.1-4.5) | 0.2 (0.0-0.4) | 12.7 (10.1-15.2) | 19.7 (15.4-23.7) | 5.1 (3.0-7.2) |
| Denmark | 0.1 (0.1-0.2) | 0.2 (0.1-0.4) | 0.0 (0.0-0.1) | 4.1 (3.5-4.9) | 4.9 (3.9-6.1) | 3.3 (2.4-4.3) | 2.9 (2.3-3.6) | 4.3 (3.2-5.5) | 1.3 (0.8-2.0) | 13.4 (12.2-14.7) | 15.6 (13.6-17.6) | 10.8 (8.9-12.8) |
| Estonia | 0.0 (0.0-0.1) | 0.0 (0.0-0.1) | 0.0 (0.0-0.1) | 14.8 (12.4-17.2) | 18.1 (14.8-21.2) | 8.6 (5.8-11.7) | 1.8 (1.0-2.8) | 2.0 (0.8-3.3) | 1.4 (0.6-2.4) | 31.0 (28.1-33.9) | 36.8 (33.0-40.4) | 19.8 (16.0-24.1) |
| Finland | 0.0 (0.0-0.1) | 0.1 (0.0-0.2) | 0.0 (0.0-0.1) | 4.5 (3.2-5.6) | 4.3 (2.4-6.0) | 4.7 (2.9-6.1) | 1.2 (0.7-1.9) | 1.7 (0.8-2.8) | 0.7 (0.1-1.3) | 12.2 (9.9-14.1) | 12.8 (9.3-15.8) | 11.6 (8.7-14.1) |
| France | 0.0 (0.0-0.0) | 0.0 (0.0-0.0) | 0.0 (0.0-0.1) | 2.5 (1.9-3.3) | 2.2 (1.3-3.2) | 3.0 (1.8-4.3) | 0.7 (0.3-1.1) | 0.6 (0.1-1.1) | 0.7 (0.2-1.3) | 7.8 (6.1-9.4) | 7.2 (5.1-9.6) | 8.5 (6.1-10.5) |
| Germany | 0.4 (0.3-0.6) | 0.6 (0.4-0.9) | 0.1 (0.0-0.1) | 12.7 (11.8-13.6) | 15.8 (14.4-17.1) | 8.9 (7.6-10.2) | 12.0 (11.0-13.1) | 17.1 (15.4-18.7) | 5.7 (4.6-6.8) | 34.6 (33.6-35.8) | 41.3 (40.1-42.9) | 26.2 (24.2-28.3) |
| Greece | 0.0 (0.0-0.1) | 0.0 (0.0-0.0) | 0.0 (0.0-0.2) | 11.0 (5.5-16.4) | 7.2 (0.0-14.3) | 14.7 (6.0-23.7) | 1.1 (0.0-2.8) | 1.0 (0.0-3.6) | 1.3 (0.0-3.1) | 24.8 (16.6-32.7) | 19.8 (6.3-33.1) | 29.7 (19.3-39.8) |
| Hungary | 0.1 (0.0-0.3) | 0.2 (0.0-0.6) | 0.0 (0.0-0.1) | 4.9 (3.0-6.7) | 6.2 (3.1-9.0) | 3.7 (1.3-6.0) | 2.4 (0.7-4.0) | 4.1 (0.9-7.1) | 0.8 (0.0-1.7) | 13.9 (10.5-17.1) | 16.9 (11.6-21.6) | 11.0 (6.6-15.6) |
| Ireland | 0.1 (0.0-0.1) | 0.1 (0.0-0.2) | 0.0 (0.0-0.1) | 3.2 (2.3-4.2) | 4.8 (3.0-6.5) | 1.6 (0.8-2.5) | 1.3 (0.7-1.9) | 1.8 (0.8-2.9) | 0.7 (0.2-1.3) | 9.8 (8.0-11.4) | 13.7 (10.5-16.4) | 5.9 (4.0-7.7) |
| Italy | 0.0 (0.0-0.1) | 0.0 (0.0-0.1) | 0.0 (0.0-0.0) | 1.2 (0.3-2.2) | 1.1 (0.0-2.6) | 1.3 (0.0-2.9) | 1.2 (0.4-2.0) | 1.9 (0.5-3.3) | 0.5 (0.0-1.1) | 6.7 (3.4-9.4) | 7.1 (2.3-11.5) | 6.2 (1.7-10.2) |
| Latvia | 0.1 (0.0-0.2) | 0.1 (0.0-0.3) | 0.0 (0.0-0.0) | 4.1 (2.2-6.0) | 6.1 (2.8-9.2) | 1.7 (0.3-3.4) | 1.9 (0.8-3.2) | 3.5 (1.4-5.8) | 0.1 (0.0-0.5) | 13.4 (10.0-16.6) | 19.3 (14.0-24.3) | 6.6 (2.9-10.0) |
| Montenegro | 0.0 (0.0-0.0) | 0.0 (0.0-0.1) | 0.0 (0.0-0.0) | 4.1 (2.5-6.0) | 5.5 (2.7-8.6) | 2.7 (0.9-4.8) | 0.8 (0.0-1.7) | 1.7 (0.1-3.3) | 0.0 (0.0-0.2) | 15.6 (12.0-19.1) | 20.0 (14.3-25.5) | 11.2 (6.8-15.5) |
| Netherlands | 0.2 (0.0-0.3) | 0.3 (0.1-0.6) | 0.0 (0.0-0.0) | 4.6 (3.3-5.9) | 7.7 (5.3-10.0) | 1.4 (0.4-2.5) | 3.9 (2.7-5.3) | 6.6 (4.4-9.0) | 1.1 (0.2-2.1) | 15.9 (13.2-18.6) | 24.0 (20.0-27.9) | 7.6 (4.4-10.9) |
| Portugal | 0.0 (0.0-0.0) | 0.0 (0.0-0.0) | 0.0 (0.0-0.0) | 3.1 (2.4-3.9) | 5.0 (3.6-6.3) | 1.0 (0.5-1.7) | 0.2 (0.1-0.3) | 0.4 (0.1-0.6) | 0.0 (0.0-0.1) | 8.2 (6.9-9.6) | 12.5 (10.3-14.6) | 3.3 (2.1-4.7) |
| Romania | 0.0 (0.0-0.1) | 0.0 (0.0-0.1) | 0.0 (0.0-0.0) | 17.5 (14.6-20.5) | 22.0 (18.0-26.3) | 12.7 (8.9-16.8) | 1.3 (0.5-2.2) | 2.4 (0.8-4.0) | 0.3 (0.0-0.8) | 29.4 (26.4-33.1) | 35.9 (32.0-40.7) | 22.7 (17.9-27.9) |
| Serbia | 0.0 (0.0-0.0) | 0.0 (0.0-0.1) | 0.0 (0.0-0.0) | 4.1 (2.7-5.5) | 5.2 (2.9-7.7) | 2.9 (1.4-4.5) | 1.2 (0.5-2.0) | 2.0 (0.6-3.4) | 0.4 (0.0-0.9) | 12.7 (9.8-15.5) | 15.9 (11.5-20.4) | 9.4 (5.8-12.7) |
| Slovenia | 0.0 (0.0-0.2) | 0.1 (0.0-0.3) | 0.0 (0.0-0.0) | 16.9 (12.7-21.4) | 21.9 (15.6-28.8) | 11.5 (6.4-16.6) | 2.3 (0.4-4.2) | 4.1 (0.4-7.8) | 0.3 (0.0-0.8) | 31.2 (26.3-36.5) | 39.7 (32.9-47.5) | 21.9 (15.2-28.5) |
| Spain | 0.0 (0.0-0.0) | 0.0 (0.0-0.0) | 0.0 (0.0-0.0) | 6.3 (3.6-9.0) | 7.3 (3.5-10.6) | 5.4 (1.3-9.7) | 0.0 (0.0-0.1) | 0.0 (0.0-0.1) | 0.0 (0.0-0.2) | 17.2 (12.5-21.7) | 16.6 (10.4-21.8) | 17.8 (10.9-25.5) |
| Sweden | 0.0 (0.0-0.0) | 0.0 (0.0-0.0) | 0.0 (0.0-0.0) | 2.1 (1.4-2.8) | 2.5 (1.3-3.6) | 1.6 (0.9-2.4) | 0.3 (0.1-0.5) | 0.5 (0.2-0.8) | 0.2 (0.0-0.3) | 6.2 (4.7-7.5) | 7.2 (5.0-9.2) | 4.9 (3.4-6.4) |
| United Kingdom | 0.6 (0.2-1.0) | 1.0 (0.3-1.7) | 0.1 (0.0-0.2) | 14.5 (12.4-16.4) | 17.4 (14.4-20.0) | 10.8 (8.4-13.1) | 4.8 (3.4-6.2) | 6.6 (4.4-9.0) | 2.4 (1.2-3.7) | 29.0 (26.6-31.7) | 32.7 (29.4-36.1) | 24.3 (21.1-28.1) |

^1^ Crude and utilizable protein intake was predicted by a linear mixed model, with protein intake (g/kg/d) as dependent variable, and the ratio of reported energy intake over required energy intake as independent variable. The model included a random intercept and random slope for subjects and adjusted for sex, country, and body mass index. An exponential covariance structure was applied to correctly consider consecutive and non-consecutive measurement days. Protein intake was predicted when the ratio of reported energy intake over required energy intake was equal to 1, i.e. energy intake equals energy requirement.

^2^ Observed protein intakes as observed in the food consumption data, including substantial percentages of under-reporting.

## Supplementary table 7

Supplemental table 7. Contribution (%) of crude protein intake of each meal to the daily protein intake.

|  | Before breakfast | Breakfast | Snack between breakfast and lunch | Lunch | Snack between lunch and dinner | Dinner | Snack after dinner | Un-classified |
| --- | --- | --- | --- | --- | --- | --- | --- | --- |
| Austria | 1 | 18 | 5 | 32 | 8 | 32 | 4 | 0 |
| Belgium | 0 | 14 | 2 | 32 | 5 | 42 | 4 | 0 |
| Bosnia Herzegovina | 1 | 32 | 2 | 39 | 3 | 20 | 2 | 1 |
| Croatia | 0 | 10 | 11 | 39 | 14 | 23 | - | 3 |
| Cyprus | 16 | 12 | 6 | 32 | 6 | 26 | 2 | - |
| Czech Republic | 1 | 12 | 7 | 38 | 16 | 24 | 2 | 0 |
| Denmark | - | 19 | 24 | 42 | 0 | 3 | 6 | 5 |
| Estonia | 0 | 22 | 2 | 32 | 4 | 34 | 4 | 1 |
| Finland | 1 | 18 | 4 | 28 | 10 | 24 | 15 | 1 |
| France | 0 | 12 | 1 | 44 | 3 | 39 | 1 | - |
| Germany | 1 | 11 | 12 | 25 | 16 | 31 | 5 | - |
| Greece | 0 | 14 | 53 | 1 | 4 | 27 | 1 | - |
| Hungary | - | 19 | 5 | 40 | 7 | 27 | 2 | 1 |
| Ireland | - | 16 | 2 | 27 | 3 | 44 | 6 | 1 |
| Italy | 0 | 10 | 2 | 37 | 3 | 47 | 1 | - |
| Latvia | - | 20 | 5 | 34 | 5 | 31 | 4 | 2 |
| Montenegro | 1 | 28 | 3 | 42 | 4 | 21 | 1 | 0 |
| Netherlands | 0 | 16 | 4 | 24 | 6 | 43 | 7 | 0 |
| Portugal | 1 | 13 | 3 | 39 | 9 | 32 | 2 | 0 |
| Romania | 1 | 24 | 2 | 38 | 3 | 30 | 2 | 0 |
| Serbia | 1 | 27 | 4 | 41 | 3 | 22 | 2 | 0 |
| Slovenia | 0 | 16 | 10 | 43 | 6 | 21 | 2 | 1 |
| Spain | - | 17 | 5 | 39 | 5 | 32 | 1 | - |
| Sweden | - | 18 | - | 30 | - | 40 | - | 12 |
| United Kingdom | - | - | - | - | - | - | - | 100 |

Empty cells (-) indicate that the respective meal moment was not utilized in the dataset.

## Supplementary table 8

Supplemental table 8. Crude indispensable amino acid intake in mg/kg body weight/day (median [Interquartile range]).

|  | Isoleucine | Leucine | Lysine | SAA | AAA | Threonine | Tryptophan | Valine | Histidine |
| --- | --- | --- | --- | --- | --- | --- | --- | --- | --- |
| Amino acid requirement (mg/kg/d) | 20 | 39 | 30 | 15 | 25 | 15 | 4 | 26 | 10 |
| Country |  |  |  |  |  |  |  |  |  |
| Austria | 45 [35-59] | 76 [58-97] | 64 [47-86] | 32 [25-42] | 79 [60-100] | 35 [27-47] | 12 [9-15] | 54 [42-70] | 26 [20-34] |
| Belgium | 47 [36-60] | 78 [59-98] | 70 [53-92] | 33 [26-42] | 78 [60-99] | 37 [29-48] | 12 [9-15] | 55 [42-70] | 28 [22-36] |
| Bosnia and Herzegovina | 43 [34-54] | 71 [56-89] | 63 [47-80] | 32 [25-39] | 72 [57-89] | 34 [27-44] | 10 [8-13] | 51 [40-63] | 26 [20-32] |
| Croatia | 50 [38-64] | 82 [64-106] | 76 [57-99] | 36 [28-47] | 83 [63-106] | 41 [32-53] | 12 [9-16] | 59 [46-76] | 30 [23-39] |
| Cyprus | 52 [38-65] | 85 [63-107] | 80 [56-104] | 36 [27-46] | 85 [64-107] | 42 [30-54] | 12 [9-16] | 62 [45-78] | 31 [23-39] |
| Czech Republic | 50 [38-64] | 83 [63-106] | 73 [54-94] | 37 [28-46] | 85 [64-108] | 41 [31-52] | 13 [10-16] | 60 [45-76] | 30 [22-38] |
| Denmark | 49 [40-61] | 82 [67-101] | 72 [58-91] | 34 [28-42] | 82 [67-102] | 39 [32-49] | 12 [10-15] | 59 [48-73] | 29 [24-36] |
| Estonia | 41 [30-54] | 67 [50-88] | 62 [45-84] | 28 [21-38] | 67 [50-88] | 33 [24-44] | 10 [8-13] | 49 [36-65] | 24 [18-32] |
| Finland | 50 [39-63] | 83 [65-105] | 76 [58-99] | 35 [27-44] | 84 [65-106] | 40 [31-51] | 12 [10-16] | 60 [47-75] | 29 [22-37] |
| France | 55 [43-70] | 91 [71-115] | 82 [63-105] | 39 [30-49] | 93 [72-118] | 44 [34-56] | 13 [11-17] | 66 [51-83] | 33 [25-41] |
| Germany | 38 [29-49] | 65 [49-83] | 55 [41-73] | 28 [21-36] | 66 [51-85] | 31 [23-40] | 10 [8-13] | 46 [35-59] | 23 [17-30] |
| Greece | 42 [31-54] | 70 [52-90] | 60 [44-81] | 29 [22-38] | 70 [53-93] | 33 [24-44] | 10 [8-14] | 50 [38-65] | 23 [18-31] |
| Hungary | 50 [39-63] | 82 [64-103] | 74 [56-93] | 36 [28-45] | 83 [64-104] | 40 [31-50] | 12 [9-15] | 59 [46-75] | 29 [23-37] |
| Ireland | 51 [41-63] | 86 [70-106] | 78 [62-97] | 37 [30-45] | 85 [69-105] | 42 [34-52] | 13 [10-15] | 61 [50-76] | 31 [25-38] |
| Italy | 52 [41-67] | 85 [68-109] | 78 [59-102] | 37 [29-47] | 88 [70-113] | 42 [33-54] | 13 [10-16] | 62 [49-78] | 31 [24-40] |
| Latvia | 48 [36-63] | 79 [58-103] | 75 [55-100] | 33 [24-43] | 78 [58-102] | 39 [29-52] | 12 [9-15] | 58 [43-75] | 29 [21-38] |
| Montenegro | 45 [36-57] | 75 [61-95] | 65 [50-84] | 33 [27-42] | 76 [62-95] | 36 [29-46] | 11 [9-14] | 53 [43-67] | 26 [21-34] |
| Netherlands | 46 [36-58] | 77 [61-97] | 66 [51-85] | 33 [26-41] | 79 [62-99] | 37 [29-46] | 12 [9-15] | 55 [43-69] | 27 [21-34] |
| Portugal | 57 [41-77] | 92 [68-124] | 89 [63-124] | 41 [30-56] | 92 [68-123] | 47 [34-64] | 14 [10-18] | 66 [49-90] | 33 [24-45] |
| Romania | 43 [29-60] | 71 [49-98] | 64 [42-91] | 31 [21-43] | 72 [49-98] | 35 [24-48] | 10 [7-15] | 51 [35-71] | 25 [17-36] |
| Serbia | 51 [39-65] | 84 [65-107] | 75 [55-98] | 37 [29-47] | 85 [66-108] | 41 [31-53] | 12 [10-16] | 60 [47-78] | 30 [23-39] |
| Slovenia | 42 [30-58] | 71 [50-95] | 61 [43-88] | 31 [22-41] | 71 [51-95] | 34 [24-47] | 10 [8-14] | 50 [36-69] | 25 [18-35] |
| Spain | 44 [35-56] | 72 [58-91] | 68 [53-86] | 31 [25-39] | 72 [58-89] | 36 [28-46] | 11 [8-13] | 52 [42-66] | 27 [20-33] |
| Sweden | 57 [45-69] | 94 [75-113] | 88 [69-109] | 40 [32-49] | 93 [76-114] | 46 [37-57] | 14 [11-17] | 68 [55-82] | 34 [27-41] |
| United Kingdom | 41 [33-52] | 70 [56-87] | 62 [48-78] | 30 [23-37] | 70 [56-87] | 34 [27-42] | 10 [8-13] | 51 [41-64] | 25 [20-31] |

## Supplementary table 9

Supplemental table 9. Proportion (%) of meals with a limiting amino acid, or not containing any protein, by meal moment and country. Breakfast, lunch and dinner accounted for 82% of total protein intake. In-between meals are left out from this figure. The aromatic amino acids and threonine were never limiting and therefore not shown in this graph. The United Kingdom is not included in this graph since data on meal moments was not available. Some food items were not categorized as part of a meal and were instead labeled as 'Unclassified'. These food items are left out of this table.

| Country |  | Proportion (%) of meals with a limiting amino acid | | | | | | | No protein (%) | Total # of meals |
| --- | --- | --- | --- | --- | --- | --- | --- | --- | --- | --- |
|  | Meal | Lysine | Isoleucine | Sulphur amino acids | Leucine | Tryptophan | Valine | Histidine |  |  |
| Austria | Breakfast | 30.77 | 0.03 | 1.22 | 0.03 | 0 | 0 | 0 | 6.54 | 3855 |
|  | Lunch | 14.17 | 0.03 | 1.74 | 0.16 | 0.03 | 0 | 0 | 2.47 | 3727 |
|  | Dinner | 14.04 | 0.03 | 1.48 | 0.13 | 0.05 | 0 | 0 | 4.30 | 3981 |
| Belgium | Breakfast | 38.41 | 0 | 0.97 | 0.10 | 0 | 0 | 0 | 7.03 | 2062 |
|  | Lunch | 12.57 | 0.09 | 1.06 | 0.05 | 0.05 | 0.05 | 0 | 2.29 | 2180 |
|  | Dinner | 6.69 | 0 | 1.10 | 0.04 | 0 | 0 | 0 | 0.88 | 2271 |
| Bosnia and Herzegovina | Breakfast | 15.73 | 0 | 0.44 | 0.06 | 0 | 0 | 0 | 1.82 | 1596 |
|  | Lunch | 9.07 | 0 | 0.78 | 0.13 | 0 | 0 | 0 | 0.58 | 1543 |
|  | Dinner | 16.09 | 0.22 | 6.20 | 0.22 | 0 | 0 | 0 | 8.78 | 1355 |
| Croatia | Breakfast | 15.43 | 0.02 | 0.88 | 0.02 | 0 | 0 | 0 | 26.60 | 4557 |
|  | Lunch | 7.71 | 0.02 | 1.64 | 0.06 | 0 | 0 | 0 | 5.76 | 4952 |
|  | Dinner | 12.55 | 0 | 1.77 | 0 | 0.04 | 0 | 0 | 14.63 | 5203 |
| Cyprus | Breakfast | 19.72 | 0.23 | 0.70 | 0 | 0 | 0 | 0 | 12.44 | 426 |
|  | Lunch | 7.76 | 0.24 | 6.82 | 0.24 | 0 | 0 | 0 | 2.35 | 425 |
|  | Dinner | 12.12 | 0 | 2.56 | 0 | 0 | 0 | 0 | 6.29 | 429 |
| Czech Republic | Breakfast | 42.58 | 0.04 | 0.32 | 0 | 0 | 0.20 | 0 | 8.85 | 2487 |
|  | Lunch | 6.39 | 0.13 | 0.70 | 0.03 | 0 | 0.13 | 0 | 2.93 | 3007 |
|  | Dinner | 15.99 | 0.30 | 1.47 | 0 | 0 | 1.43 | 0 | 10.66 | 3001 |
| Denmark | Breakfast | 16.68 | 0.06 | 0.71 | 0 | 0 | 0 | 0 | 4.33 | 10764 |
|  | Lunch | 2.13 | 0 | 0.18 | 0 | 0.05 | 0 | 0 | 0.60 | 11101 |
|  | Dinner | 11.39 | 0.83 | 5.54 | 0.01 | 1.44 | 0 | 0.013 | 49.21 | 7621 |
| Estonia | Breakfast | 8.63 | 0.10 | 0.69 | 0.10 | 0.03 | 0 | 0.026 | 3.09 | 3916 |
|  | Lunch | 5.27 | 0 | 1.27 | 0.03 | 0.03 | 0 | 0 | 1.30 | 3697 |
|  | Dinner | 5.92 | 0.28 | 1.18 | 0.18 | 0.10 | 0 | 0.026 | 2.31 | 3888 |
| Finland | Breakfast | 6.71 | 0.04 | 0.66 | 0 | 0.04 | 0 | 0 | 3.09 | 2264 |
|  | Lunch | 2.00 | 0.11 | 0.32 | 0 | 0 | 0 | 0 | 0.16 | 1904 |
|  | Dinner | 1.71 | 0 | 0.43 | 0 | 0 | 0 | 0 | 0.12 | 1640 |
| France | Breakfast | 47.16 | 0.06 | 0.54 | 0 | 0 | 0 | 0 | 10.26 | 4824 |
|  | Lunch | 4.77 | 0.12 | 0.60 | 0.06 | 0.02 | 0 | 0 | 0.87 | 5032 |
|  | Dinner | 6.31 | 0.06 | 0.68 | 0.08 | 0 | 0 | 0 | 1.10 | 5185 |
| Germany | Breakfast | 30.28 | 0 | 0.54 | 0.01 | 0.01 | 0.02 | 0 | 15.91 | 13057 |
|  | Lunch | 12.55 | 0.02 | 2.01 | 0.76 | 0.10 | 0.05 | 0 | 11.13 | 16903 |
|  | Dinner | 13.31 | 0.04 | 0.86 | 0.54 | 0.23 | 0.09 | 0 | 6.56 | 18179 |
| Greece | Breakfast | 29.73 | 0 | 0.62 | 0.21 | 0.21 | 0 | 0 | 14.97 | 481 |
|  | Lunch | 25.81 | 1.61 | 8.06 | 0 | 1.61 | 0 | 0 | 45.16 | 62 |
|  | Dinner | 12.47 | 0.23 | 1.85 | 0 | 0 | 0 | 0 | 3.46 | 433 |
| Hungary | Breakfast | 20.42 | 0.10 | 0.62 | 0 | 0 | 0.10 | 0 | 4.90 | 960 |
|  | Lunch | 5.16 | 0.11 | 1.05 | 0.11 | 0 | 0.11 | 0 | 0.74 | 950 |
|  | Dinner | 18.48 | 0.21 | 0.63 | 0 | 0 | 0.11 | 0 | 4.22 | 947 |
| Ireland | Breakfast | 25.42 | 0.50 | 0.63 | 0 | 0 | 0 | 0 | 1.11 | 4591 |
|  | Lunch | 14.05 | 0.43 | 0.84 | 0.13 | 0 | 0 | 0 | 0.13 | 3942 |
|  | Dinner | 4.75 | 0.29 | 0.47 | 0 | 0 | 0 | 0 | 0.05 | 4438 |
| Italy | Breakfast | 35.85 | 0.37 | 0.59 | 0 | 0 | 0 | 0 | 9.39 | 1353 |
|  | Lunch | 10.13 | 0.22 | 0.94 | 0.22 | 0 | 0 | 0 | 0.36 | 1382 |
|  | Dinner | 7.92 | 0 | 0.43 | 0.07 | 0 | 0 | 0.144 | 0.94 | 1389 |
| Latvia | Breakfast | 9.24 | 0 | 1.03 | 0.09 | 0 | 0 | 0 | 10.68 | 2144 |
|  | Lunch | 5.71 | 0.09 | 3.76 | 0.42 | 0.09 | 0 | 0 | 1.25 | 2154 |
|  | Dinner | 8.86 | 0.18 | 3.03 | 0.50 | 0.23 | 0 | 0 | 2.89 | 2213 |
| Montenegro | Breakfast | 19.16 | 0 | 0.87 | 0 | 0 | 0 | 0 | 1.43 | 1263 |
|  | Lunch | 7.30 | 0 | 1.05 | 0.15 | 0 | 0 | 0 | 0.23 | 1329 |
|  | Dinner | 16.64 | 0.09 | 2.09 | 0 | 0 | 0 | 0 | 3.14 | 1148 |
| Netherlands | Breakfast | 29.79 | 0 | 1.22 | 0 | 0.04 | 0 | 0 | 3.37 | 2702 |
|  | Lunch | 27.80 | 0 | 0.46 | 0.08 | 0 | 0 | 0 | 1.20 | 2586 |
|  | Dinner | 3.57 | 0 | 0.28 | 0.18 | 0 | 0 | 0 | 0.07 | 2828 |
| Portugal | Breakfast | 27.95 | 0.16 | 0.96 | 0.19 | 0.07 | 0 | 0 | 5.27 | 5731 |
|  | Lunch | 4.00 | 0.03 | 1.41 | 1.32 | 0.03 | 0 | 0 | 0.27 | 5972 |
|  | Dinner | 6.47 | 0.02 | 2.35 | 4.23 | 0.07 | 0 | 0.034 | 0.56 | 5906 |
| Romania | Breakfast | 19.13 | 0.07 | 2.91 | 0 | 0 | 0 | 0 | 10.98 | 1375 |
|  | Lunch | 12.21 | 0 | 1.25 | 0 | 0 | 0 | 0 | 1.94 | 1441 |
|  | Dinner | 13.53 | 0.21 | 2.48 | 0 | 0 | 0 | 0 | 4.55 | 1449 |
| Serbia | Breakfast | 18.01 | 0.19 | 0.89 | 0.05 | 0 | 0 | 0 | 3.09 | 2138 |
|  | Lunch | 7.82 | 0 | 1.82 | 0.19 | 0.05 | 0 | 0 | 0.37 | 2148 |
|  | Dinner | 19.84 | 0.25 | 2.04 | 0.10 | 0.05 | 0 | 0 | 6.98 | 2006 |
| Slovenia | Breakfast | 31.74 | 0 | 3.24 | 0 | 0 | 0 | 0 | 10.32 | 649 |
|  | Lunch | 6.40 | 0.28 | 1.56 | 0.57 | 0 | 0 | 0 | 1.42 | 703 |
|  | Dinner | 18.08 | 0.15 | 3.09 | 0.77 | 0 | 0 | 0 | 8.19 | 647 |
| Spain | Breakfast | 13.33 | 0.29 | 0.48 | 0 | 0 | 0 | 0 | 4.60 | 1043 |
|  | Lunch | 3.69 | 0.19 | 1.23 | 0.09 | 0 | 0 | 0 | 0 | 1056 |
|  | Dinner | 3.55 | 0.29 | 0.96 | 0.10 | 0 | 0 | 0 | 1.15 | 1043 |
| Sweden | Breakfast | 9.22 | 0.02 | 0.40 | 0 | 0 | 0 | 0.040 | 1.24 | 4981 |
|  | Lunch | 4.31 | 0.29 | 0.94 | 0.35 | 0 | 0 | 0 | 0.44 | 4552 |
|  | Dinner | 3.63 | 0.04 | 0.49 | 0.10 | 0.02 | 0 | 0.020 | 0.26 | 4929 |
